# Typology of persons with severe mental disorders

**DOI:** 10.1186/1471-244X-13-137

**Published:** 2013-05-11

**Authors:** Marie-Josée Fleury, Guy Grenier, Jean-Marie Bamvita, Jacques Tremblay

**Affiliations:** 1 Department of Psychiatry, McGill University, Douglas Hospital Research Centre, 6875 LaSalle Blvd, Montreal, Quebec H4H 1R3, Canada; 2 Douglas Hospital Research Centre, 6875 LaSalle Blvd, Montreal, Quebec, H4H 1R3, Canada

## Abstract

**Background:**

Persons with severe mental disorders (PSMD) form a highly heterogeneous group. Identifying subgroups sharing similar PSMD profiles may help to develop treatment plans and appropriate services for their needs. This study seeks to establish a PSMD typology by looking at individual characteristics and the amount and adequacy of help received.

**Methods:**

The study recruited a sample of 352 persons located in south-western Montreal (Quebec, Canada). Cluster analysis was used to create a PSMD typology.

**Results:**

Analysis yielded five clusters: 1. highly functional older women with mood disorders, receiving little help from services; 2. middle-aged men with diverse mental disorders and alcohol abuse, receiving insufficient and inadequate help; 3. middle-aged women with serious needs, mood and personality disorders and suicidal tendencies, living in autonomous apartments, and receiving ample but inadequate help; 4. highly educated younger men with schizophrenia, living in autonomous apartments, and receiving adequate help; and 5. older poorly educated men with schizophrenia, living in supervised apartments, with ample help perceived as adequate. Marked differences were found between men and women, between users diagnosed with schizophrenia and others, and between persons living in supervised or autonomous apartments.

**Conclusion:**

Our study highlights the existence of parallel subgroups among PSMD related to their socio-demographic status, clinical needs and service-use profiles, which could be used to focus more appropriate interventions. For mental health service planning, it demonstrates the relevance of focusing on individuals showing critical needs who are affected by multiple mental disorders (especially when associated with alcohol abuse), and often find help received as less adequate.

## Background

Persons with severe mental disorders (PSMD) are heavy users of health services [1] who form a highly heterogeneous group, varying widely in terms of clinical and socio-demographic characteristics, needs, and service utilization [2]. Classification systems like DSM-IV provide a detailed clinical picture of mental disorders but cannot anticipate needs, service use, or outcomes [3]. For example, women commonly report a more benign illness course, a lower level of disability, better social integration, and greater service use than men affected by similar severe mental disorders [4]. Marital status, income, urban or rural living conditions, access to health services, and co-morbidity are others factors reported in the literature as modulating service utilization and outcomes among PSMD [5].

Identifying, describing and validating various subgroups sharing similar clinical and socio-demographic characteristics may help to develop treatment plans and appropriate services for their needs [3,6,7]. Cluster analysis is a useful method to organize and establish a typology of mental health services user [1,6]. Using clusters, PSMD can be included in subgroups characterized by a different profile correlated with clinical and socio-demographic variables and patterns of service use [3,8]. From a sample of 2,447 PSMD, Herman & Mowbray [1] have identified six clusters labeled “Poorest Functioning/High Health Needs,” “Psychotic,” “Suicidal/Aggressive,” “Mentally Ill Substance Abuser,” “Demoralized” and “Best Functioning.” Based on a set of 467 individuals hospitalized with a dual diagnosis of severe mental and substance abuse disorders, Luke et al. [9] found seven clusters labeled “Best Functioning,” “Unhealthy Alcohol Abuse,” “Functioning Alcohol Abuse,” “Drug Abuse,” “Functioning Polyabuse,” “Criminal Polyabuse” and “Unhealthy Polyabuse.” From a sample of 203 individuals with schizophrenia treated in the community, Lora et al. [10] identified four clusters 1) mild severity of illness and low service use; 2) more severe disability, low severity in psychiatric symptoms, moderate family burden and more intensive community service use; 3) serious disability and severe positive symptoms, distressing family burden and intensive hospital and community service use; and 4) very severe disability, prominent negative symptoms, moderate family burden, frequent hospital resource use, and low community service use. Other studies have identified clusters among frequent users of in-patient services [11,12], psychiatric in-patients hospitalized for the first time [6], homeless with mental and general medical disorders [13], and PSMD using resources for homeless persons [7].

Variables used in those cluster analyses usually include age, gender, marital status, residential situation, social support, diagnosis, severity of symptoms, frequency of hospitalization, or health service use during a period. To our knowledge, however, no study using cluster analysis has identified profiles of PSMD according to the severity of need, the amount of help received from services or relatives, and adequacy of help to patient needs. In terms of needs assessment, some studies have found a link between serious needs and symptom severity, lower social functioning and poorer quality of life [14,15]. The amount and adequacy of help from services and relatives were also associated with severity of need. Usually, help received is adequate in case of moderate needs, but no adequate for serious needs [16,17]. The inclusion of these original variables in cluster analysis could lead to the development of new profiles among PSMD.

Using a cluster analysis, and with a view to improving service planning, this study aims to create a PSMD typology based on clinical, socio-demographic, and needs characteristics, and amount and adequacy of help received from relatives and services.

## Methods

### Study design and sample selection criteria

This cross-sectional study involved PSMD who were under continued treatment at the Douglas Mental Health University Institute (DMHUI) located in the southwest of Montreal City, in Quebec, Canada. This metropolitan area covers two local health networks, encompassing a population of 258,000. The DMHUI offers specialized mental healthcare (i.e., second- and third-line services). Two health and social service centres, the South-West-Verdun Health and Social Service Centre (SWVHSSC) and the Dorval-Lachine-LaSalle Health and Social Service Centre (DLLHSSC), created through the merger of a general hospital (with no psychiatric department), local community service centres and nursing homes, provide primary mental health services.

The sample for the study was selected within the registry of patients who received treatment and follow-up by the DMHUI exclusively or both the DMHUI and HSSC. To participate in the study, participants had to be aged between 18 and 65, diagnosed with severe mental disorders diagnosis criteria 147 (schizophrenia and other psychotic disorders) or 161 (mood disorders) according to DSM-IV [18] and live within the catchment area. They also had to agree to the research team reviewing their medical records, and refer the team to their principal case manager who would then be asked to complete a questionnaire on their community functioning. PSMD showing severe mental retardation or receiving an involuntary psychiatric treatment as decided by a judiciary board or having a history of hospitalization or emergency room visits in the three months prior to the initial interview were considered unable to complete the questionnaires and were thus excluded from the study.

Recruitment involved several strategies: posters at the MHUI, and at the two HSSC for patient self-referral; recruitment by outpatient clinical staff; and information sessions or letters sent to explain the project to providers or housing resource staff. The data collection period ran from December 2008 to September 2010. A team of trained clinical professionals conducted the interviews under the close supervision of a research coordinator. Except for self-referrals, candidates were contacted by their case manager, who gauged their interest for the study and subsequently referred potential participants to the research team. Two 90-minute interviews at one week interval were conducted with each participant. Each participant had to sign a consent form after the study was described to them. The study protocol was approved by the DMHUI (07-35), SWHSCC and DLLHSCC ethics boards.

### Study measurement instruments

Data was collected using six questionnaires, administered both in English and French, and from participants’ medical records at the MHI: 1) The Montreal Assessment of Needs Questionnaire (MANQ; Additional file 1) was derived from the Camberwell Assessment of Needs Questionnaire (CAN), which is one of the most reliable instruments for needs assessment [19]. The CAN evaluates 22 needs domains by taking into account their number and their severity, along with the amount of help received from relatives and services, and the adequacy of help, in terms of quality (right kind of help) and quantity (amount of help received). For the MANQ, four domains (stress adaptation, social exclusion, involvement in treatment decisions, and job integration), were added based on their pertinence for patient recovery [20], for a total of 26 domains. Unlike the CAN, which measures patients’ needs on an ordinal scale, the MANQ uses analog scales, ranging from 0 to 10. The aim of this change was to improve variability in the data without compromising precisions. The MANQ also includes socio-demographic data (e.g. age, housing), socio-economic aspects (e.g. source of income), and profiles service use (professional consulted) by patients.

The MANQ was used previously to identify factors associated with needs of PSMD [21], adequacy of help [22], and amount of help received from services and relatives [23]. In another study, currently under submission, the MANQ has been validated using various analyses (such as factor analyses, Cronbach Alpha, test-retest and interrater reliability testing, and corresponding values on the MANQ for each category of the CAN). Participants were asked to complete the MANQ and CAN during the same interview. Overall, validation analyses showed that the two instruments were comparable. Cronbach Alpha in the MANQ ranged from 0.70 to 0.73 (versus 0.68 to 0.71 for the CAN). The factor structure was quite identical and the kappa coefficients in test-retest and interrater reliability testing with the MANQ were quite similar to those found in previous studies with the CAN for each of the needs domains.

Other standardized instruments were 2) the Alcohol Use Disorders Identification Test (AUDIT), measuring the degree of dependency and risky alcohol consumption [24]; 3) the Drug Abuse Screening Test-20 (DAST-20), evaluating drug abuse and consequences [25]; 4) the Social Provisions Scale (SPS), exploring patient level of integration and social support [26]; 5) the Multnomah Community Ability Scale (MCAS), assessing community functioning [27]; and 6) the Service Utilisation questionnaire (SUQ) derived from the Canadian Community Health Survey questionnaire evaluating type and frequency of service use for mental health needs over a 12-month period preceding patient recruitment [28]. All participants completed the questionnaire with the help of the interviewer, except the MCAS, which the principal case manager filled out. Participant medical records were used to compile supportive clinical data on diagnoses according to the DSM-IV and history of suicide attempts.

#### Analyses

Participant typology was carried out by means of a TwoStep cluster analysis using SPSS Statistics 20.0 package [29,30]. It is coined “TwoStep” because it proceeds in two steps: a pre-cluster of participants into small sub-classes, followed by the final cluster of sub-classes into an appropriate number of classes based on specific statistical tests, or into a number of classes determined by the statistician based on the best interpretability of the model. Cluster analysis is similar to the Latent Class analysis (LCA) run with SAS package. The two techniques were introduced in the 1950s [31,32]. Both techniques are similar in that they have a common purpose: to unmask latent clusters of subjects with similar profiles. In addition, they both generate mutually exclusive and exhaustive classes, use an objective technique to determine the number of classes (Bayesian Information Criterion for LCA or Schwartz Bayesian criteria for TwoStep Clustering), and work with categorical and continuous variables. Cluster analysis has the advantage of being better known and more widely used than LCA.

The TwoStep cluster analysis was performed according to the following classic steps: Variables selected were organized in continuous and categorical variables. Categorical variables included gender, education, housing, schizophrenia, personality disorders, suicide attempts, mood disorders, and anxiety disorders. Continuous variables were age, severity of need, adequacy of help, amount of help from services or relatives, and AUDIT, MCAS, DAST-20 and SPS total scores. Categorical variables were entered first, followed by continuous variables. The Log-likelihood method was used to determine inter-subject distance and specific classification of participants. The first model was carried out using Schwartz Bayesian criteria, yielding seven classes. We subsequently applied many different models using different numbers of predetermined classes. We set the final number of clusters at five, according to their overall contribution to inter-class homogeneity as determined by the diagnostic test of model improvement.

## Results

Overall, we approached 437 candidates; 116 (27%) lived in an intermediary resource, 61 (14%) in a foster home, and 254 (58%) in other housing types (e.g. autonomous or supervised apartment). A total of 352 individuals (80.5%) agreed to take part in the study, and 85 declined (19.5%). Refusals were compared to participants for age and housing type. No statistically significant difference was found for (1) intermediary residence participants (Chi-square: 5.999 [P = 0.199]); (2) foster home participants (Chi-square: 4.482 [P = 0.482]); (3) or other housing type (Chi-square: 3,229 [P = 0.665]). Participants were also compared as to gender distribution (total sample), and no statistically significant difference was found (Chi-square: 1,210 [P = 0.271]).

Table 1 shows socio-demographic, socio-economic, and clinical characteristics while Table 2 displays continuous variables. The mean age for the 352 participants was 46.5 (SD: 10.9), with 186 men (53%) and 166 women (47%). Most participants were French-speaking (64%). Only 13% were in a relationship. Sixty-six percent were on welfare. The majority had not completed a post-secondary education (65%). Sixty-one percent lived in autonomous apartments. The most prevalent mental health disorders were mood disorders (40%) and schizophrenia (38%). The mean number of perceived needs was 8 per user (S.D. 4.4), for an overall severity score of 50.7 (S.D. 33.4). Users received more help from services than relatives. Adequacy of help received was average with a mean of 6.2 (S.D. 2.5) per user. Previous studies gave more detailed descriptions of the socio-demographic, socio-economical and clinical characteristics of patients who took part in this study [21-23].

**Table 1:** Socio-demographic, socio-economic and clinical variables (N = 352)

**Categories**	**Sub-categories**	**Variables**		**n**	**%**
**Socio-demographic variables**	**Age categories**	20-29 years old		37	10.5
		30-39 years old		51	14.5
		40-49 years old		103	29.3
		50-59 years old		121	34.4
		60-70 years old		40	11.4
	**Gender**	Men		186	52.8
		Women		166	47.2
	**Spoken language**	French		225	63.9
		English		73	20.7
		French/English		8	2.3
		Others		46	13.1
	**Civil status**	Single/Never married		251	71.3
		Partnered /Married/Remarried		46	13.1
		Separated /Divorced/Widowed		55	15.6
**Socio-economic variables**	**Source of income**	Welfare		234	66.5
		Other sources		118	33.5
	**Education**	Primary school		39	11.1
		Secondary school		190	54.0
		College		72	20.5
		University		51	14.5
	**Type of housing**	Apartment		212	60.2
		Intermediary resource		57	16.2
		Foster home		52	14.8
		Temporary housing		14	4.0
		Supervised housing		16	4.5
		Homeless		1	0.3
**Clinical variables**	**Mental health disorders**	Schizophrenia		134	38.1
		Mood disorders		142	40.3
		Schizophrenia spectrum disorders		45	12.8
		Delusion and other psychotic disorders		33	9.4
		Anxiety disorders		41	11.6
		Dependencies	Alcohol	13	3.7
			Drug	22	6.3
			Multiple	41	11.6
		Personality disorders		97	27.6
		Mild mental retardation		45	12.8
		History of suicide attempts		112	31.8

**Table 2:** Distribution of main continuous variables in the sample (N = 352)

	**Mean**	**SD**	**Range (min - max)**
Age	46.5	10.9	19-66
MCAS score	65.2	9.7	28-83^a^
AUDIT score	5.9	6.5	0-35^b^
DAST-20 score	2.7	3.3	0-15^b^
SPS score	70.6	8.1	0-94^a^
Number of diagnoses	1.7	0.9	0-4
Number of perceived needs	8.0	4.4	0-26
Global severity of perceived needs	50.7	33.4	0-180
Amount of help from relatives	22.7	24.0	0-134
Level of help received from relatives	2.3	2.4	0-10
Amount of help from services	33.9	22.7	0-124
Level of help received from services	3.9	2.5	0-10
Adequacy of help received from relatives and services	6.2	2.5	0-10
a: Higher score = more favorable b: Higher score = less favorable			

Five clusters of participants were identified (Table 3). The sub-sample sizes in the five clusters ranged from 83 (24%) in Cluster 5 to 55 (16%) in the Cluster 1. Only three participants were automatically eliminated from classification. The cluster model retained fifteen variables based on their importance for the characterization of participants and their discriminative results between clusters. This process led to the elimination of DAST-20 and SPS scores, which had yielded highly similar mean scores among clusters.

**Table 3:** Cluster analysis of patients with mental disorders (N = 352; eliminated: 3 = 0.9%)

		**Class 1 (N = 55; 15.8%)**		**Class 2 (N = 65; 18.6%)**		**Class 3 (N = 76; 21.8%)**		**Class 4 (N = 70; 20.1%)**		**Class 5 (N = 83; 23.8%)**		**Combined (N = 349; 100.0%)**	
Need seriousness [Mean (SD)]		46.5	34.0	59.8	35.4	63.1	34.0	43.4	26.8	43.0	30.7	51.1	33.2
Adequacy of help [Mean (SD)]		6.0	2.6	5.7	2.3	5.6	2.3	6.2	2.3	7.2	2.6	6.2	2.5
Amount of help from services [Mean (SD)]		29.2	18.8	30.1	16.3	32.9	23.6	31.7	20.7	43.8	27.6	34.1	22.8
Amount if help from relatives [Mean (SD)]		27.6	19.2	21.2	19.2	28.4	31.1	25.8	25.4	13.5	18.8	22.9	24.0
Audit score [Mean (SD)]		4.1	4.7	7.4	7.4	5.6	8.1	6.5	5.8	5.9	5.6	6.0	6.5
MCAS score [Mean (SD)]		69.0	8.3	66.8	8.2	65.7	8.6	66.4	9.3	59.9	11.1	65.2	9.7
Age categories [n(%)]	<30 years old	5	13.5	4	10.8	7	18.9	10	27.0	11	29.7	37	100.0
	30-39 years old	7	13.7	12	23.5	12	23.5	13	25.5	7	13.7	51	100.0
	40-49 years old	11	10.9	25	24.8	22	21.8	20	19.8	23	22.8	101	100.0
	50-59 years old	20	16.7	16	13.3	27	22.5	26	21.7	31	25.8	120	100.0
	>59 years old	12	30.0	8	20.0	8	20.0	1	2.5	11	27.5	40	100.0
Gender [n(%)]	Women	55	33.3	0	0.0	68	41.2	12	7.3	30	18.2	165	100.0
	Men	0	0.0	65	35.3	8	4.3	58	31.5	53	28.8	184	100.0
Education [n(%)]	Secondary school or -	25	11.0	41	18.1	48	21.2	31	13.7	82	36.1	227	100.0
	College/University	30	24.6	24	19.7	28	23.0	39	32.0	1	0.8	122	100.0
Type of housing [n(%)]	Autonomous	39	18.4	45	21.2	71	33.5	57	26.9	0	0.0	212	100.0
	Supervised	16	11.7	20	14.6	5	3.6	13	9.5	83	60.6	137	100.0
Schizophrenia [n(%)]		2	1.5	3	2.3	0	0.0	60	45.1	68	51.5	133	100.0
Personality disorders		1	1.0	18	18.6	52	53.6	10	10.3	16	16.5	97	100.0
History of prior suicide attempt [n(%)]		0	0.0	21	18.8	49	43.8	19	17.0	23	20.5	112	100.0
Mood disorders		42	29.8	51	36.2	47	33.3	1	0.7	0	0.0	141	100.0
Anxiety disorders		8	19.5	21	51.2	7	17.1	3	7.3	2	4.9	41	100.0
Labels		Highly functional older women with mood disorders, receiving little help from services		Middle-aged men with diverse mental disorders and alcohol abuse, and receiving insufficient and inadequate help		Middle-aged women with serious needs, mood and personality disorders, and suicidal tendencies, living in autonomous apartments, and receiving ample but inadequate help		Highly educated younger men with schizophrenia, living in autonomous apartments, receiving adequate help		Older poorly educated men with schizophrenia, living in supervised apartments with ample help perceived as adequate			

Cluster 1 comprised women (100%) performing well on MCAS score and having a higher proportion of individuals aged over 60. It ranked second in terms of the amount of help from relatives, and also had the second-highest ratio of participants with college or university education and anxiety disorders. The most frequent diagnosis in this cluster was mood disorders (N = 42 or 76.3%). This group showed the lowest AUDIT score and received the least amount of help from services. We labeled this cluster “Highly functional older women with mood disorders, receiving little help from services.”

Cluster 2 comprised men (100%) mostly aged between 40 and 49, with the highest AUDIT score and ratio of mood and anxiety disorders. This cluster ranked second for severity of need, MCAS score, and ratio of persons in supervised living arrangements, and with personality disorders. In terms of amount and adequacy of help received from services and relatives, Cluster 2 ranked next to last. We labeled this cluster “Middle-aged men with diverse mental disorders and alcohol abuse, receiving insufficient and inadequate help.”

Cluster 3 held mostly middle-aged women, living in autonomous apartments, with the highest proportion of personality disorders and most suicide attempts. Individuals in this cluster reported the highest mean of severity of need and the highest amount of help from relatives. They ranked second in terms of amount of help received from services, and proportion of persons with primary or secondary education and mood disorders. This group had the lowest score of help adequacy, and proportion of individuals living in supervised apartments. No individuals with schizophrenia were part of this cluster. We labeled this cluster “Middle-aged women with serious needs, mood and personality disorders and suicide tendencies, living in autonomous apartments and receiving ample but inadequate help.”

Cluster 4 had the highest percentage of younger individuals (less than 40 years old), predominantly men, with college or university education. They ranked second as to AUDIT score and adequacy of help, and ratio of persons with schizophrenia and living in autonomous apartments. This cluster, which comprised the lowest proportion of individuals over 60, was labeled “Highly educated younger men with schizophrenia, living in autonomous apartments, and receiving adequate help.”

Cluster 5 subsumed primarily persons aged 50 or more with primary or secondary education, living in supervised apartments. It had the highest rates of schizophrenia, amount of help received from services, and adequacy of help. This cluster ranked second on history of suicide attempts, had the lowest scores in terms of severity of need, help received from relatives and MCAS score. It also showed the lowest proportion of individuals with a college or university degree and anxiety disorders. No person with a mood disorders or living in autonomous apartments was part of this cluster, which we labeled “Older, poorly educated men with schizophrenia living in supervised apartments, with ample help from services perceived as adequate.”

Complementary characteristics of clusters on service use are displayed in Tables 4 (categorical variables) and 5 (continuous variables). In terms of healthcare professionals consulted (Table 4), patients visited most often a psychiatrist (53%). The most frequent contacts for cluster 5 patients were with family physicians and social workers while cluster 2 patients sought mainly psychiatrists and other professionals. Cluster 4 patients called most often on the services of nurses and cluster 3 patients had their most frequent contacts with psychologists. We also found the highest percentage of visits to at least one professional in the past 12 months among cluster 2 patients.

**Table 4:** Complementary characteristics of clusters of participants with mental disorders: categorical variables

	**Total sample (N = 349; 100.0%)**		**Class 1 (N = 55; 15.8%)**		**Class 2 (N = 65; 18.6%)**		**Class 3 (N = 76; 21.8%)**		**Class 4 (N = 70; 20.1%)**		**Class 5 (N = 83; 23.8%)**	
**Healthcare professionals visited in last 12 months**	**n**	**%**	**n**	**%**	**n**	**%**	**n**	**%**	**n**	**%**	**n**	**%**
Family physician	153	43.5	23	41.8	28	43.1	36	47.4	23	32.9	41	49.4
Psychiatrist	187	53.1	30	54.5	43	66.2	46	60.5	28	40.0	40	48.2
Nurse	144	40.9	18	32.7	25	38.5	34	44.7	36	51.4	30	36.1
Social worker	106	30.1	11	20.0	20	30.8	19	25.0	22	31.4	34	41.0
Psychologist	47	13.4	12	21.8	12	18.5	17	22.4	4	5.7	2	2.4
Other professionals	200	57.3	29	52.7	46	70.8	34	44.7	38	54.3	47	56.6
At least one professional	334	94.9	51	92.7	64	98.5	74	97.4	64	91.4	79	95.2

In terms of overall intensity of care received, psychiatrists are again the most-frequently-visited professionals (Table 5). As to the intensity of care received by clusters, family physicians, psychiatrists and nurses are most frequently visited by cluster 5 patients, social workers by cluster 2 patients, and psychologists by cluster 4 patients. Cluster 2 patients have the highest mean of total frequency of visits to healthcare professionals.

**Table 5:** Complementary characteristics of clusters of participants with mental disorders: continuous variables

		**Total sample (N = 349; 100.0%)**		**Class 1 (N = 55; 15.8%)**		**Class 2 (N = 65; 18.6%)**		**Class 3 (N = 76; 21.8%)**		**Class 4 (N = 70; 20.1%)**		**Class 5 (N = 83; 23.8%)**	
		**Mean**	**SD**	**Mean**	**SD**	**Mean**	**SD**	**Mean**	**SD**	**Mean**	**SD**	**Mean**	**SD**
Frequency of visit to specific healthcare professionals in the previous 12 months	Family physician	2.8	7.4	1.9	2.3	2.8	5.9	1.5	1.4	3.5	8.9	4.0	11.5
	Psychiatrist	3.5	6.9	2.3	5.0	3.0	5.8	2.0	3.2	3.0	6.0	6.4	10.4
	Nurse	2.9	7.1	2.0	4.7	3.3	7.3	2.2	5.2	2.0	5.7	4.6	10.0
	Social worker	1.4	5.0	1.0	2.6	2.0	5.3	1.5	5.6	0.5	1.7	1.9	6.9
	Psychologist	0.2	2.4	0.0	0.2	0.3	1.6	0.0	0.0	0.5	3.4	0.4	3.6
Frequency of visits to healthcare professionals		5.0	2.7	5.2	3.2	5.4	2.6	4.9	2.4	5.1	3.1	4.5	2.5
Number of professionals visited		2.9	1.1	2.8	1.2	3.2	1.0	2.9	1.0	2.7	1.2	2.8	1.1

## Discussion

This study was designed to develop a PSMD typology, based on socio-demographic and clinical characteristics, severity of needs, help received from services or relatives, and adequacy of help. Its purpose was to use PSMD clusters to facilitate mental health-care planning efforts. Five profiles emerged from analysis. There were marked difference between men and women, between users diagnosed with schizophrenia and other mental disorders, and between persons living in autonomous and in supervised apartments.

Two clusters (1 and 3) were more closely associated with women. Of note is that schizophrenia is virtually absent from those two clusters. In our sample, men showed the most schizophrenia cases. The main differences between Clusters 1 and 3 were age, clinical variables, and education level, along with help received from services. Cluster 1 included mostly older women with a single diagnosis – generally mood disorders – and few serious needs. Women in this cluster were better educated and more functional according to the MCAS score. Conversely, Cluster 3 included for the most part middle-aged women with personality disorders, though one third also showed mood disorders, and a history of suicide attempts. Cluster 3 patients had more serious needs, which may explain both the higher amount of help from services and the lower adequacy of help. According to the literature, individuals affected by personality disorders have more numerous and serious needs than those affected by other severe mental disorders [16]. Moreover, clusters with a majority of women received the most help from relatives, independently of severity of needs, and presented a lower incidence of alcohol abuse according to the AUDIT score, compared to clusters made up mostly of men – which is consistent with findings from the literature [7,33,34].

Two clusters (4 and 5) included more individuals with schizophrenia and few serious needs. Men were overrepresented in those clusters. Mood and anxiety disorders were virtually absent from clusters 4 and 5 and, conversely, schizophrenia was virtually absent from the three other clusters. The main difference between clusters 4 and 5 was the housing type. Cluster 4 included mainly those living in autonomous apartments while cluster 5 was composed exclusively of persons in supervised apartments. Patients in Cluster 5 were also older, less functional, and less educated and received more help from services. Patients in Cluster 4 were more autonomous and received more help from relatives.

According to some authors, co-morbidity with mental disorders appears to be the norm [35,36]. In two of the five clusters patients suffered from more than one mental disorders. In clusters 2 and 3, co-morbidity with mood, anxiety and personality disorders, and history of suicide attempts was especially frequent. Personality disorders and suicide attempts seem to be especially related. In all clusters, the number of individuals with personality disorders is almost identical to that of individuals with a history of suicide attempts. Clusters 2 and 3 showed notable similitude with clusters identified by Herman & Mowbray [1]. As the “Mentally Ill Substance Abuser” group (1), cluster 2 was mainly constituted of men with alcohol abuse problems as indicated by the AUDIT score (mean of 7.4, whereas 8 to 15 is considered at moderate risk of harm). Cluster 3 shared similar patterns with both the “Suicidal/Aggressive” and the “Demoralized” groups [1] – two clusters where women are overrepresented. As in the “Suicidal/Aggressive” group, our cluster 2 showed a high frequency of personality disorders and suicide attempts. Moreover, while the mean AUDIT score was low (5.6), the high standard deviation (8.1) indicated that a number of individuals in cluster 2 had a serious alcohol abuse problem. Furthermore, as in the “Demoralized” group, cluster 3 had a high ratio of mood disorders and suicidal behaviour. Another similitude with Herman and Mowbray typology [1] was that most individuals in clusters 2 and 3 lived in autonomous apartments. Persons in cluster 3 received more help from services and much more from relatives. Finally, needs were particularly more serious in clusters 2 and 3, and help was inadequate. Co-morbidity of disorders tended to be more chronic than unique mental disorders, and treatment was less effective [37].

Marked differences also exist between the clusters in terms of housing type. While all users in cluster 5 lived in supervised apartments, conversely almost all users in cluster 3 lived in autonomous apartments. The only clinical variable that can explain those differences is community functioning as indicated by the very low MCAS scores in cluster 5. Lower functional skills, associated with negative symptoms of schizophrenia, usually result in lower levels of education [38] and constitute an obstacle to social integration. Living in supervised apartments promotes satisfaction, which explains the higher level of adequacy of help. Age could be another explanation. Individuals living in supervised apartments were more numerous in clusters 1 and 5, which comprised more individuals aged 50 or over.

Adequacy of help appears to be associated, not with the amount of help received, but with the severity of needs and the presence of co-morbidity. Clusters 1, 4 and 5 where help was perceived to be more adequate were also those where needs were the least serious and where individuals usually presented only one diagnosis (usually mood disorders in cluster 1, schizophrenia in clusters 4 and 5). Conversely, the severity of needs was extremely high in clusters 2 and 3, where most individuals had multiple mental disorders. The amount of help received from services or relatives was insufficient to meet the needs of those individuals, who often presented suicidal behaviour and suffered concurrently from mood, personality and anxiety disorders and, in the case of cluster 2, substance abuse problems.

Finally, significant differences exist between clusters in terms of type and number of professionals consulted and frequency of visits with these professionals. Patients in clusters 2 and 3 saw the greatest number of professionals and had the most visits in a 12-month period. It is probable that users from these clusters had more reasons to see professionals because they suffer from multiple mental disorders and show more acute needs [35]. Mood and anxiety disorders, with or co-occurring abuse disorders, are the most significant predictor of service use [39-42]. Moreover, clusters 2 and 3 are mainly constituted of patients aged between 30 and 49 years old. Middle-age patients are the heaviest users of mental health services [43]. Furthermore, the high proportion of visits to healthcare professionals by cluster 1 patients seems to confirm that women seek professional help more often than men independently of the severity of their mental condition or needs [5,41,44-46]. Finally, the high frequency of contacts with family physicians, psychiatrists and nurses among cluster 5 patients makes sense because users in this latter group are older and live in supervised apartments, which means that they are more likely to be regularly visited by a health-care professional than people living in autonomous apartments [47].

The main limitation of this study was the number of variables that could be introduced in the model via cluster analyses. This would indicate that this analysis remains at a rather exploratory level [33]. There are also limitations to external validity as our results may not readily be generalized to other samples or populations. Specifically, the proportions of patients 30 years old or less and over 59 years old in our sample were relatively small.

## Conclusion

Cluster analyses revealed five PSMD clusters presenting considerable heterogeneity in terms of socio-demographic characteristics, clinical variables, and amount and adequacy of help received. Our study highlights the relevance for mental health service planning of focusing on individuals affected by multiple mental disorders and with clearly critical severity of needs who also find that the help they receive is less than adequate. These are generally middle-aged men or women living in autonomous apartments. As comorbid alcohol abuse is rampant among PSMD, greater collaboration between professionals and programs within the health-care system, especially with regard to mental disorders and substance abuse, should lead to more timely and proper care. Specifically, caregivers should consider integrated programs or shared-care initiatives during the intervention care planning period. Finally, more outreach and awareness programs are needed to identify needs and facilitate access to mental health services for younger and older patients since these sub-groups are generally more reticent to use such services.

## Abbreviations

AUDIT: Alcohol Use Disorders Identification Test; CAN: Camberwell Assessment of Need; DAST-20: Drug Abuse Screening Test-20; DLLHSCC: Dorval-Lachine-LaSalle Health and Social Service Centre; DMHUI: Douglas Mental Health University Institute; DSM-IV: Diagnostic and Statistic Manual of Mental Disorders, 4th Edition; HSSC: Health and Social Service Centres; MANQ: Montreal Assessment of Needs Questionnaire; MCAS: Multnomah Community Ability Scale; PSMD: Persons with severe mental disorders; SPS: Social Provision Scale; SWVHSSC: South-West-Verdun Health and Social Service Centre.

## Competing interests

The authors declare that they have no competing interests.

## Authors’ contributions

MJF and GG designed the study. JMB carried out the statistical analyses with assistance from JT. MJF and GG wrote the article. All authors have read and approved the final manuscript.

## Pre-publication history

The pre-publication history for this paper can be accessed here:

http://www.biomedcentral.com/1471-244X/13/137/prepub

## Supplementary Material

Additional file 1 Montreal Assessment of Needs Questionnaire (MANQ). Click here for file
