# Supplementary material for: Typology of persons with severe mental disorders
Source: BMC Psychiatry. 2013 May 11;13:137. doi: 10.1186/1471-244X-13-137 (PMC3655095; doi:10.1186/1471-244X-13-137)
Supplement: Additional file 1 — Montreal Assessment of Needs Questionnaire (MANQ). [file 1471-244X-13-137-S1.doc]

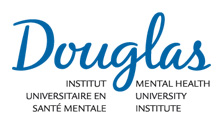


**ANNEX 1**

| **MONTREAL ASSESSMENT OF NEEDS QUESTIONNAIRE (MANQ)**  Research version (cr1.6) |
| --- |

| ***Revised version for the research project :***  ***Déterminants de l’adéquation des services aux besoins des personnes***  ***avec troubles importants de santé mentale dans les RIS***  *(Fleury, M.J.; Tremblay, J.; Piat, M.; Perreault, M.; Caron; J., IRSC, 2007-2011)* |
| --- |

# *For more information:*

Marie-Josée Fleury Jacques Tremblay

Flemar@douglas.mcgill.ca trejac@douglas.mcgill.ca

Douglas Mental Health University Institute

6875, LaSalle Blvd., Verdun, Canada H4H 1R3

Tel.: (514) 761-6131

Fax: (514) 762-3049

| **HOW TO USE THE RESEARCH VERSION OF THE**  **MONTREAL ASSESSMENT OF NEEDS QUESTIONNAIRE (MANQ)** |
| --- |

The research version of the MANQ can be used to rate the views of users. Before using the MANQ, please read the entire schedule, including the examples, which give guidance about ratings.

The current scale includes 26 topics, each with four sections. Sections A, B and C, item 23-26 and open questions on appreciation of services have been added by the research team from the original Camberwell Assessment of Needs* (CAN) to complete the user profile.

The team also added a continuous scale to reflect diversity of user opinions. Responses can be marked by filling a box anywhere on the response line.

**SECTION 1** Asks if there is currently a problem in each topic area **in the last month**.

If the person rates NO PROBLEM or NOT KNOWN, the interviewer must go to the next topic. If not, the respondent must complete the entire section.

If the second box is checked, then the interviewer must ask all questions of the topic.

For each topic suggested questions to ask are in *italics*.

**SECTION 2** Asks about help received from informal carers **during the last month**.

**SECTION 3** Asks about help received and needed from paid staff **during the last month**.

**SECTION 4** Asks about the appropriateness of the care given, and about the user’s rating of satisfaction with the amount of care received.

For more precisions, consult the “answer cart” at page 60.

* CAN has been designed at PRiSM by Michael Phelan, Mike Slade, Graham Dunn, Frank Holloway, Geraldine Strathdee, Graham Thronicroft and Til Wykes. Summary sheets and further copies of the CAN may be obtained from PRiSM, Institute of Psychiatry, De Crespigny Park, London SE5 8AZ. Phone: 071 919 2610 Fax: 071 277 1462

TABLE OF CONTENTS

A. SOCIO-DEMOGRAPHIC INFORMATION 4

B. SERVICE USE 6

1. ACCOMMODATION 7

2. FOOD 9

3. LOOKING AFTER THE HOME 11

4. SELF CARE 13

5. DAYTIME ACTIVITIES 15

6. PHYSICAL HEALTH 17

7. PSYCHOTIC SYMPTOMS 19

8. INFORMATION (ABOUT CONDITION AND TREATMENT) 21

9. PSYCHOLOGICAL DISTRESS 23

10. SAFETY TO SELF 25

11. SAFETY TO OTHERS 27

12. ALCOHOL 29

13. DRUGS 31

14. COMPANY 33

15. INTIMATE RELATIONSHIPS 35

16. SEXUAL EXPRESSION 37

17. CHILDCARE 39

18. BASIC EDUCATION 41

19. TELEPHONE 43

20. TRANSPORT 45

21. MONEY 47

22. SOCIAL BENEFITS 49

23. WORK 51

24. ADAPTATION TO DAILY STRESS 53

25 SOCIAL EXCLUSION 55

26 INVOLVEMENT IN DECISIONS CONCERNING TREATMENT……………….…….…57

C. MAIN SUPPORT CONSULTED 59

D. ANSWER CART……………………………………………………………………………..60

| A. SOCIO-DEMOGRAPHIC INFORMATION | | Assessments -Checkresponse | Code |
| --- | --- | --- | --- |
|  |  | *User* |  |
| Age | Specify age : _________________________________________ |  | 2310 |
| Sex | Male | **🞅** | 2311 |
|  | Female | **🞅** | 2312 |
| Current relationship / marital status | Single/never married | **🞅** | 2313 |
|  | De facto union (living with partner for at least 2 years) | **🞅** | 2314 |
|  | Married/remarried | **🞅** | 2315 |
|  | Separated | **🞅** | 2316 |
|  | Divorced | **🞅** | 2317 |
|  | Widowed (deceased spouse) | **🞅** | 2318 |
|  | Other (specify): _____________________________________ | **🞅** | 2319 |
| Education | No schooling (illiterate) | **🞅** | 2320 |
|  | Primary | **🞅** | 2321 |
|  | Secondary | **🞅** | 2322 |
|  | College | **🞅** | 2323 |
|  | University (specify) : _______________________________ | **🞅** | 2324 |
|  | Other (specify) : ___________________________________ | **🞅** | 2325 |
| Source of income | Paid employment | **🞅** | 2326 |
|  | Government pension | **🞅** | 2327 |
|  | Social benefits (social security) | **🞅** | 2328 |
|  | Food pension | **🞅** | 2329 |
|  | Unemployment insurance | **🞅** | 2330 |
|  | Money shared with spouse/partner | **🞅** | 2331 |
|  | Company pension | **🞅** | 2332 |
|  | Other (specify): _____________________________________ | **🞅** | 2333 |

| Residence | Unsupervised apartment | **🞅** | 2334 | | |
| --- | --- | --- | --- | --- | --- |
|  | Supervised apartment | **🞅** | 2335 | | |
|  | Low-rent housing (ex. : H.L.M. or O.S.B.L) | **🞅** | 2336 | | |
|  | Room or boarding house | **🞅** | 2337 | | |
|  | Family residence | **🞅** | 2338 | | |
|  | Intermediary residence | **🞅** | 2339 | | |
|  | Homeless | **🞅** | 2340 | | |
|  | Other (specify): _____________________________________ | **🞅** | 2341 | | |
| Children | Specify number of children : _____________________________ |  | 2342 | | |
|  | Full custody | **🞅** | 2343 | | |
|  | Shared custody | **🞅** | 2344 | | |
|  | Custody held by other relative | **🞅** | 2345 | | |
|  | Adoption | **🞅** | 2346 | | |
|  | Other (specify): _____________________________________ | **🞅** | 2347 | | |
| Tobacco use | Non-smoker | **🞅** | 2348 | | |
|  | Smoker | **🞅** | 2349 | | |
|  | For how long : _____________________________ |  | 2350 | | |
|  | How many cigarettes/day : ____________________________ |  | 2351 | | |
| Ethnicbackground | Country of birth : ____________________________________ | | | 2352 |  |
|  | Nationality(s) : _______________________________________ | | | 2353 |  |
|  | Cultural background of ancestors : _______________________ | | | 2354 |  |
|  | Ethno-cultural self-identification : ______________________ | | | 2355 |  |
|  | Mother tongue : ___________________________________ | | | 2356 |  |
|  | Religion : ___________________________________________  Please rate the importance of spirituality in your life.  **□□□□□□□□□□□** **□** NOT KNOWN  No importance High importance | | | 2357  2358 |  |

## B. SERVICE USE

*List staff consulted* ***during the last month.***

*Specify their profession, their affiliated organization, the type of services offered by this organization, the length of the user/staff relationship (in months), the frequency of contacts (per month) as well as the modality and places of meeting*

| **Support consulted** | **Profession** | **Organization** | **Type of services** | **Since when?** | **Frequency of contacts / month** | | **Place of meeting** |
| --- | --- | --- | --- | --- | --- | --- | --- |
|  | *ex. social worker, nurse* | *e.g. CSSS (CLSC mandate or hosp.), Douglas (psychiatric hosp)* | *e.g. housing, external clinic, community services* |  | **Face to face** | **Over the phone** | *e.g. services, at home or public space* |
|  |  |  |  |  |  |  |  |
|  |  |  |  |  |  |  |  |
|  |  |  |  |  |  |  |  |
|  |  |  |  |  |  |  |  |
|  |  |  |  |  |  |  |  |
|  |  |  |  |  |  |  |  |
|  |  |  |  |  |  |  |  |

*N.B. Verify if user has consulted a psychiatrist, a family doctor (specify if consulted for physical health or mental health or both), a hospital-based worker (CHSGS, CHPSY), or staff from a CLSC or other organizations on CSSS territory (e.g. community-based organizations). Verify if emergency services were used (for what reasons)*

| **Number of relative/friends helping** | **Nature of the relationship**  **(e.g. mother, spouse, brother, sister, etc.)** | **Frequency of contacts/month** | |
| --- | --- | --- | --- |
| **Relatives : ___________** |  | **Face to**  **face** | **Over the**  **phone** |
|  |  |  |  |
|  |  |  |  |
|  |  |  |  |
|  |  |  |  |
|  |  |  |  |
| **Close**  **friends : ________** | **Nature of the relationship**  **(e.g.: peer support, childhood friend, etc.)** |  |  |
|  |  |  |  |
|  |  |  |  |
|  |  |  |  |
|  |  |  |  |
|  |  |  |  |

| 1. ACCOMMODATION |  | |
| --- | --- | --- |
|  | Assessments | |
|  | *User* |  |
| DOES THE PERSON LACK A CURRENT PLACE TO LIVE?*What kind of place do you live in?* *What sort of place is it?* | Can 0101 |  |
|  |  |  |
| Please rate importance of current problem.  **□□□□□□□□□□□** **□** NOT KNOWN  No problem Very serious problem  NO PROBLEM e.g. Home is completely suitable.  VERY SERIOUS PROBLEM e.g. Home is absolutely unsuitable.   IF RATING IS *NO PROBLEM* OR *NOT KNOWN*, GO TO ITEM 2. | | |
| LEVEL OF HELP RECEIVED (FRIENDS OR RELATIVES)HOW MUCH HELP DOES THE PERSON RECEIVE FROM FRIENDS OR RELATIVES WITH THEIR ACCOMMODATION? | Can 0103 |  |
|  |  |  |
| **□□□□□□□□□□□** **□** NOT KNOWN  None Very high help  NONE e.g. Receives nothing.  VERY HIGH HELP e.g. Living with relative because own accommodation is unsatisfactory. | | |
| LEVEL OF HELP RECEIVED (SERVICES)HOW MUCH HELP DOES THE PERSON RECEIVE FROM LOCAL SERVICES WITH THEIR ACCOMMODATION? | Can 0105 |  |
|  |  |  |
| **□□□□□□□□□□□** **□** NOT KNOWN  None Very high help  NONE e.g. Receives nothing.  VERY HIGH HELP e.g. Being rehoused, in living group, room or boarding house, family residence or other supervised housing. | | |
| **LEVEL OF HELP NEEDED (SERVICES)** HOW MUCH HELP DOES THE PERSON NEED FROM LOCAL SERVICES WITH THEIR ACCCOMMODATION? | Can 0107 |  |
|  |  |  |
| **□□□□□□□□□□□** **□** NOT KNOWN  None Very high help  NONE e.g. Receives nothing.  VERY HIGH HELP e.g. Being rehoused, in living group, room or boarding home, family residence or other supervised housing. | | |

|  |  | |
| --- | --- | --- |
| APPROPRIATENESS OF CARE (SERVICES)DOES THE PERSON RECEIVE THE RIGHT TYPE OF HELP WITH THEIR ACCOMMODATION? | Can 0109 |  |
|  |  |
| **QUANTITY**  **□□□□□□□□□□□** **□** NOT KNOWN  Not at all Entirely satisfied  satisfied | | |
| APPROPRIATENESS OF CARE (SERVICES)DOES THE PERSON RECEIVE THE RIGHT TYPE OF HELP WITH THEIR ACCOMMODATION? | Can 0111 |  |
|  |  |
| **QUALITY**  **□□□□□□□□□□□** **□** NOT KNOWN  Not at all Entirely satisfied  satisfied | | |
| USER’S PERSPECTIVE OF SERVICES REQUIRED | | |
| Thinking back about services received with respect to your accommodation, tell us in a few words what you most appreciated. (Write down respondent’s spontaneously given information)  In your experience, what could be improved? (Write down respondent’s spontaneously given information) | | |

| 2. FOOD | Assessments | |
| --- | --- | --- |
|  | *User* |  |
| DOES THE PERSON HAVE DIFFICULTY IN GETTING ENOUGH TO EAT?*What kind of food do you eat?**Are you able to prepare your own meals and do your own shopping?* | Can 0201 |  |
|  |  |  |
| Please rate importance of current problem.  **□□□□□□□□□□□** **□** NOT KNOWN  No problem Very serious problem  NO PROBLEM e.g. Meals are completely suitable.  VERY SERIOUS PROBLEM e.g. Meals are absolutely unsuitable. IF RATING IS *NO PROBLEM* OR *NOT KNOWN*, GO TO ITEM 3. | | |
| **LEVEL OF HELP RECEIVED (FRIENDS OR RELATIVES)** HOW MUCH HELP DOES THE PERSON RECEIVE FROM FRIENDS OR RELATIVES WITH GETTING ENOUGH TO EAT? | Can 0203 |  |
|  |  |  |
| **□□□□□□□□□□□** **□** NOT KNOWN  None Very high help  NONE e.g. No help needed. VERY HIGH HELP e.g. All meals are provided. | | |
| **LEVEL OF HELP RECEIVED (SERVICES)** HOW MUCH HELP DOES THE PERSON RECEIVE FROM LOCAL SERVICES WITH GETTING ENOUGH TO EAT? | Can 0205 |  |
|  |  |  |
| **□□□□□□□□□□□** **□** NOT KNOWN  None Very high help  NONE e.g. Receives nothing. VERY HIGH HELP e.g. All meals are provided. | | |
| **LEVEL OF HELP NEEDED (SERVICES)** HOW MUCH HELP DOES THE PERSON NEED FROM LOCAL SERVICES WITH GETTING ENOUGH TO EAT? | Can 0207 |  |
|  |  |  |
| **□□□□□□□□□□□** **□** NOT KNOWN  None Very high help  NONE e.g. Receives nothing. VERY HIGH HELP e.g. All meals are provided. | | |

| APPROPRIATENESS OF CARE (SERVICES)DOES THE PERSON RECEIVE THE RIGHT TYPE OF HELP WITH GETTING ENOUGH TO EAT? | Can 0209 |  |
| --- | --- | --- |
|  |  |
| **QUANTITY**  **□□□□□□□□□□□** **□** NOT KNOWN  Not at all Entirely satisfied  satisfied | | |
| APPROPRIATENESS OF CARE (SERVICES)DOES THE PERSON RECEIVE THE RIGHT TYPE OF HELP WITH GETTING ENOUGH TO EAT? | Can 0211 |  |
|  |  |
| **QUALITY**  **□□□□□□□□□□□** **□** NOT KNOWN  Not at all Entirely satisfied  satisfied | | |
| USER’S PERSPECTIVE OF SERVICES REQUIRED | | |
| Thinking back about services received in getting enough to eat, tell us in a few words what you most appreciated. (Write down respondent’s spontaneously given information)  In your experience, what could be improved? (Write down respondent’s spontaneously given information) | | |

| 3. LOOKING AFTER THE HOME | Assessments | |
| --- | --- | --- |
|  | *User* |  |
| DOES THE PERSON HAVE DIFFICULTY LOOKING AFTER THEIR HOME?*Are you able to look after your home?**Does anyone help you?* | Can 0301 |  |
|  |  |  |
| Please rate importance of current problem.  **□□□□□□□□□□□** **□** NOT KNOWN  No problem Very serious problem IF RATING IS *NO PROBLEM* OR *NOT KNOWN*, GO TO ITEM 4. | | |
| LEVEL OF HELP RECEIVED (FRIENDS OR RELATIVES)HOW MUCH HELP DOES THE PERSON RECEIVE FROM FRIENDS OR RELATIVES WITH LOOKING AFTER THE HOME? | Can 0303 |  |
|  |  |  |
| **□□□□□□□□□□□** **□** NOT KNOWN  None Very high help  NONE e.g. No help needed. VERY HIGH HELP e.g. Supervises the person more than once a week, washes all clothes and cleans the home. | | |
| LEVEL OF HELP RECEIVED (SERVICES)HOW MUCH HELP DOES THE PERSON RECEIVE FROM LOCAL SERVICES WITH LOOKING AFTER THE HOME? | Can 0305 |  |
|  |  |  |
| **□□□□□□□□□□□** **□** NOT KNOWN  None Very high help  NONE e.g. Receives no help. VERY HIGH HELP e.g Majority of household tasks done by staff. | | |
| **LEVEL OF HELP NEEDED (SERVICES)** HOW MUCH HELP DOES THE PERSON NEED FROM LOCAL SERVICES WITH LOOKING AFTER THE HOME? | Can 0307 |  |
|  |  |  |
| **□□□□□□□□□□□** **□** NOT KNOWN  None Very high help  NONE e.g. No help needed. VERY HIGH HELP e.g Majority of household tasks done by staff. | | |

| APPROPRIATENESS OF CARE (SERVICES)DOES THE PERSON RECEIVE THE RIGHT TYPE OF HELP WITH LOOKING AFTER THE HOME? | Can 0309 |  |
| --- | --- | --- |
|  |  |
| **QUANTITY**  **□□□□□□□□□□□** **□** NOT KNOWN  Not at all Entirely satisfied  satisfied | | |
| APPROPRIATENESS OF CARE (SERVICES)DOES THE PERSON RECEIVE THE RIGHT TYPE OF HELP WITH LOOKING AFTER THE HOME? | Can 0311 |  |
|  |  |
| **QUALITY**  **□□□□□□□□□□□** **□** NOT KNOWN  Not at all Entirely satisfied  satisfied | | |
| USER’S PERSPECTIVE OF SERVICES REQUIRED | | |
| Thinking back about services received in looking after your home, tell us in a few words what you most appreciated. (Write down respondent’s spontaneously given information)  In your experience, what could be improved? (Write down respondent’s spontaneously given information) | | |

| 4. SELF CARE | Assessments | |
| --- | --- | --- |
|  | *User* |  |
| DOES THE PERSON HAVE DIFFICULTY WITH SELF CARE?*Do you have problems keeping clean and tidy?* *Do you ever need reminding? By whom?* | Can 0401 |  |
|  |  |  |
| Please rate importance of current problem.  **□□□□□□□□□□□** **□** NOT KNOWN  No problem Very serious problem IF RATING IS *NO PROBLEM* OR *NOT KNOWN*, GO TO ITEM 5. | | |
| LEVEL OF HELP RECEIVED (FRIENDS OR RELATIVES)HOW MUCH HELP DOES THE PERSON RECEIVE FROM FRIENDS OR RELATIVES WITH THEIR SELF CARE? | Can 0403 |  |
|  |  |  |
| **□□□□□□□□□□□** **□** NOT KNOWN  None Very high help  NONE e.g. No supervision.  VERY HIGH HELP e.g. Provide daily assistance with several aspects of self care. | | |
| LEVEL OF HELP RECEIVED (SERVICES)HOW MUCH HELP DOES THE PERSON RECEIVE FROM LOCAL SERVICES WITH THEIR SELF CARE? | Can 0405 |  |
|  |  |  |
| **□□□□□□□□□□□** **□** NOT KNOWN  None Very high help  NONE e.g. No supervision. VERY HIGH HELP e.g. Supervise several aspects of self care, self care skills program. | | |
| **LEVEL OF HELP NEEDED (SERVICES)** HOW MUCH HELP DOES THE PERSON NEED FROM LOCAL SERVICES WITH THEIR SELF CARE? | Can 0407 |  |
|  |  |  |
| **□□□□□□□□□□□** **□** NOT KNOWN  None Very high help  NONE e.g. No supervision. VERY HIGH HELP e.g. Supervise several aspects of self care, self care skills program. | | |

| APPROPRIATENESS OF CARE (SERVICES)DOES THE PERSON RECEIVE THE RIGHT TYPE OF HELP WITH SELF CARE? | Can 0409 |  |
| --- | --- | --- |
|  |  |
| **QUANTITY**  **□□□□□□□□□□□** **□** NOT KNOWN  Not at all Entirely satisfied  satisfied | | |
| APPROPRIATENESS OF CARE (SERVICES)DOES THE PERSON RECEIVE THE RIGHT TYPE OF HELP WITH SELF CARE? | Can 0411 |  |
|  |  |
| **QUALITY**  **□□□□□□□□□□□** **□** NOT KNOWN  Not at all Entirely satisfied  satisfied | | |
| USER’S PERSPECTIVE OF SERVICES REQUIRED | | |
| Thinking back about services received for self care, tell us in a few words what you most appreciated. (Write down respondent’s spontaneously given information)  In your experience, what could be improved? (Write down respondent’s spontaneously given information) | | |

| 5. DAYTIME ACTIVITIES | Assessments | |  |
| --- | --- | --- | --- |
|  | *User* |  | |
| DOES THE PERSON HAVE DIFFICULTY WITH REGULAR, APPROPRIATE DAYTIME ACTIVITIES?*How do you spend your day?* *Do you have enough to do?* | Can 0501 |  | |
|  |  |  |  |
| Please rate importance of current problem.  **□□□□□□□□□□□** **□** NOT KNOWN  No problem Very serious problem   IF RATING IS *NO PROBLEM* OR *NOT KNOWN*, GO TO ITEM 6. | | |  |
| LEVEL OF HELP RECEIVED (FRIENDS OR RELATIVES)HOW MUCH HELP DOES THE PERSON RECEIVE FROM FRIENDS OR RELATIVES IN FINDING OR KEEPING REGULAR AND APPROPRIATE DAYTIME ACTIVITIES? | Can 0503 |  | |
|  |  |  |  |
| **□□□□□□□□□□□** **□** NOT KNOWN  None Very high help  NONE e.g. No help needed.  VERY HIGH HELP e.g. Daily help with arranging daytime activities. | | |  |
| LEVEL OF HELP RECEIVED (SERVICES)HOW MUCH HELP DOES THE PERSON RECEIVE FROM LOCAL SERVICES IN FINDING OR KEEPING REGULAR AND APPROPRIATE DAYTIME ACTIVITIES? | Can 0505 |  | |
|  |  |  |  |
| **□□□□□□□□□□□** **□** NOT KNOWN  None Very high help  NONE e.g. No help needed.  VERY HIGH HELP e.g. Attends day hospital or day centre daily. | | |  |
| **LEVEL OF HELP NEEDED (SERVICES)** HOW MUCH HELP DOES THE PERSON NEED FROM LOCAL SERVICES IN FINDING OR KEEPING REGULAR AND APPROPRIATE DAYTIME ACTIVITIES? | Can 0507 |  | |
|  |  |  |  |
| **□□□□□□□□□□□** **□** NOT KNOWN  None Very high help  NONE e.g. No help needed.  VERY HIGH HELP e.g. Attends day hospital or day centre daily. | | | |

| APPROPRIATENESS OF CARE (SERVICES)DOES THE PERSON RECEIVE THE RIGHT TYPE OF HELP WITH DAYTIME ACTIVITIES? | Can 0509 |  |
| --- | --- | --- |
|  |  |
| **QUANTITY**  **□□□□□□□□□□□** **□** NOT KNOWN  Not at all Entirely satisfied  satisfied | | |
| APPROPRIATENESS OF CARE (SERVICES)DOES THE PERSON RECEIVE THE RIGHT TYPE OF HELP WITH DAYTIME ACTIVITIES? | Can 0511 |  |
|  |  |
| **QUALITY**  **□□□□□□□□□□□** **□** NOT KNOWN  Not at all Entirely satisfied  satisfied | | |
| USER’S PERSPECTIVE OF SERVICES REQUIRED | | |
| Thinking back about services received for your daytime activities, tell us in a few words what you most appreciated. (Write down respondent’s spontaneously given information)  In your experience, what could be improved? (Write down respondent’s spontaneously given information) | | |

| 6. PHYSICAL HEALTH | Assessments | |
| --- | --- | --- |
|  | *User* |  |
| DOES THE PERSON HAVE ANY PHYSICAL DISABILITY OR ANY PHYSICAL ILLNESS?*How well do you feel physically?**Are you getting any treatment for physical problems from your doctor?* | Can 0601 |  |
|  |  |  |
| Please rate importance of current problem.  **□□□□□□□□□□□** **□** NOT KNOWN  No problem Very serious problem IF RATING IS *NO PROBLEM* OR *NOT KNOWN*, GO TO ITEM 7. | | |
| LEVEL OF HELP RECEIVED (FRIENDS OR RELATIVES)HOW MUCH HELP DOES THE PERSON RECEIVE FROM FRIENDS OR RELATIVES FOR PHYSICAL HEALTH PROBLEMS? | Can 0603 |  |
|  |  |  |
| **□□□□□□□□□□□** **□** NOT KNOWN  None Very high help  NONE e.g. No support.  VERY HIGH HELP e.g. Daily help with going to the toilet, eating or mobility. | | |
| LEVEL OF HELP RECEIVED (SERVICES)HOW MUCH HELP DOES THE PERSON RECEIVE FROM LOCAL SERVICES FOR PHYSICAL HEALTH PROBLEMS? | Can 0605 |  |
|  |  |  |
| **□□□□□□□□□□□** **□** NOT KNOWN  None Very high help  NONE e.g. No consultation.  VERY HIGH HELP e.g. Frequent hospital appointments. Alterations to home. | | |
| **LEVEL OF HELP NEEDED (SERVICES)** HOW MUCH HELP DOES THE PERSON NEED FROM LOCAL SERVICES FOR PHYSICAL HEALTH PROBLEMS? | Can 0607 |  |
|  |  |  |
| **□□□□□□□□□□□** **□** NOT KNOWN  None Very high help  NONE e.g. No need for consultation.  VERY HIGH HELP e.g. Frequent hospital appointments. Alterations to home. | | |

| APPROPRIATENESS OF CARE (SERVICES)DOES THE PERSON RECEIVE THE RIGHT TYPE OF HELP FOR PHYSICAL PROBLEMS? | Can 0609 |  |
| --- | --- | --- |
|  |  |
| **QUANTITY**  **□□□□□□□□□□□** **□** NOT KNOWN  Not at all Entirely satisfied  satisfied | | |
| APPROPRIATENESS OF CARE (SERVICES)DOES THE PERSON RECEIVE THE RIGHT TYPE OF HELP FOR PHYSICAL PROBLEMS? | Can 0611 |  |
|  |  |
| **QUALITY**  **□□□□□□□□□□□** **□** NOT KNOWN  Not at all Entirely satisfied  satisfied | | |
| USER’S PERSPECTIVE OF SERVICES REQUIRED | | |
| Thinking back about services received with respect to your physical health problem, tell us in a few words what you most appreciated. (Write down respondent’s spontaneously given information)  In your experience, what could be improved? (Write down respondent’s spontaneously given information) | | |

| 7. PSYCHOTIC SYMPTOMS | Assessments | |
| --- | --- | --- |
|  | *User* |  |
| DOES THE PERSON HAVE ANY PSYCHOTIC SYMPTOMS?*Do you ever hear voices, or have problems with your thoughts?**Are you on any medication or injections? What is it for?* | Can 0701 |  |
|  |  |  |
| Please rate importance of current problem.  **□□□□□□□□□□□** **□** NOT KNOWN  No problem Very serious problem IF RATING IS *NO PROBLEM* OR *NOT KNOWN*, GO TO ITEM 8. | | |
| LEVEL OF HELP RECEIVED (FRIENDS OR RELATIVES) **HOW MUCH HELP DOES THE PERSON RECEIVE FROM FRIENDS OR RELATIVES FOR THESE PSYCHOTIC SYMPTOMS?** | Can 0703 |  |
|  |  |  |
| **□□□□□□□□□□□** **□** NOT KNOWN  None Very high help  NONE e.g. No support.  VERY HIGH HELP e.g. Constant supervision of medication, and help with coping strategies. | | |
| LEVEL OF HELP RECEIVED (SERVICES)HOW MUCH HELP DOES THE PERSON RECEIVE FROM LOCAL SERVICES FOR THESE PSYCHOTIC SYMPTOMS? | Can 0705 |  |
|  |  |  |
| **□□□□□□□□□□□** **□** NOT KNOWN  None Very high help  NONE e.g. No supervision.  VERY HIGH HELP e.g. Medication and 24-hour hospital care or crisis care at home. | | |
| **LEVEL OF HELP NEEDED (SERVICES)** HOW MUCH HELP DOES THE PERSON NEED FROM LOCAL SERVICES FOR THESE PSYCHOTIC SYMPTOMS? | Can 0707 |  |
|  |  |  |
| **□□□□□□□□□□□** **□** NOT KNOWN  None Very high help  NONE e.g. No supervision.  VERY HIGH HELP e.g. Medication and 24-hour hospital care or crisis care at home. | | |

| APPROPRIATENESS OF CARE (SERVICES)DOES THE PERSON RECEIVE THE RIGHT TYPE OF HELP FOR PSYCHOTIC SYMPTOMS? | Can 0709 |  |
| --- | --- | --- |
|  |  |
| **QUANTITY**  **□□□□□□□□□□□** **□** NOT KNOWN  Not at all Entirely satisfied  satisfied | | |
| APPROPRIATENESS OF CARE (SERVICES)DOES THE PERSON RECEIVE THE RIGHT TYPE OF HELP FOR PSYCHOTIC SYMPTOMS? | Can 0711 |  |
|  |  |
| **QUALITY**  **□□□□□□□□□□□** **□** NOT KNOWN  Not at all Entirely satisfied  satisfied | | |
| USER’S PERSPECTIVE OF SERVICES REQUIRED | | |
| Thinking back about services received for your psychotic symptoms, tell us in a few words what you most appreciated. (Write down respondent’s spontaneously given information)  In your experience, what could be improved? (Write down respondent’s spontaneously given information) | | |

| 8. INFORMATION (ABOUT CONDITION AND TREATMENT) | Assessments | |
| --- | --- | --- |
|  | *User* |  |
| HAS THE PERSON HAD CLEAR VERBAL OR WRITTEN INFORMATION ABOUT CONDITION AND TREATMENT?*Have you been given clear information about your medication or other treatment?**How helpful has the information been?* | Can 0801 |  |
|  |  |  |
| Please rate importance of current problem.  **□□□□□□□□□□□** **□** NOT KNOWN  No problem Very serious problem IF RATING IS *NO PROBLEM* OR *NOT KNOWN*, GO TO ITEM 9. | | |
| LEVEL OF HELP RECEIVED (FRIENDS OR RELATIVES)HOW MUCH HELP DOES THE PERSON RECEIVE FROM FRIENDS OR RELATIVES IN OBTAINING SUCH INFORMATION? | Can 0803 |  |
|  |  |  |
| **□□□□□□□□□□□** **□** NOT KNOWN  None Very high help  NONE e.g. Does not receive any information.  VERY HIGH HELP e.g. Regular liaison with doctors or self-help groups. | | |
| LEVEL OF HELP RECEIVED (SERVICES)HOW MUCH HELP DOES THE PERSON RECEIVE FROM LOCAL SERVICES IN OBTAINING SUCH INFORMATION? | Can 0805 |  |
|  |  |  |
| **□□□□□□□□□□□** **□** NOT KNOWN  None Very high help  NONE e.g. Does not receive any information.  VERY HIGH HELP e.g. Has been given detailed written information or has had specific personal education. | | |
| **LEVEL OF HELP NEEDED (SERVICES)** HOW MUCH HELP DOES THE PERSON NEED FROM LOCAL SERVICES IN OBTAINING SUCH INFORMATION? | Can 0807 |  |
|  |  |  |
| **□□□□□□□□□□□** **□** NOT KNOWN  None Very high help  NONE e.g. Does not need any information.  VERY HIGH HELP e.g. Has been given detailed written information or has had specific personal education. | | |

| APPROPRIATENESS OF CARE (SERVICES)DOES THE PERSON RECEIVE THE RIGHT TYPE OF HELP IN OBTAINING INFORMATION? | Can 0809 |  |
| --- | --- | --- |
|  |  |
| **QUANTITY**  **□□□□□□□□□□□** **□** NOT KNOWN  Not at all Entirely satisfied  satisfied | | |
| APPROPRIATENESS OF CARE (SERVICES)DOES THE PERSON RECEIVE THE RIGHT TYPE OF HELP IN OBTAINING INFORMATION? | Can 0811 |  |
|  |  |
| **QUALITY**  **□□□□□□□□□□□** **□** NOT KNOWN  Not at all Entirely satisfied  satisfied | | |
| USER’S PERSPECTIVE OF SERVICES REQUIRED | | |
| Thinking back about services received in obtaining information about your condition or treatment, tell us in a few words what you most appreciated. (Write down respondent’s spontaneously given information)    In your experience, what could be improved? (Write down respondent’s spontaneously given information) | | |

| 9. PSYCHOLOGICAL DISTRESS | Assessments | |
| --- | --- | --- |
|  | *User* |  |
| DOES THE PERSON SUFFER FROM CURRENT PSYCHOLOGICAL DISTRESS?*Have you recently felt very sad or low?**Have you felt overly anxious or frightened?* | Can 0901 |  |
|  |  |  |
| Please rate importance of current problem.  **□□□□□□□□□□□** **□** NOT KNOWN  No problem Very serious problem   IF RATING IS *NO PROBLEM* OR *NOT KNOWN*, GO TO ITEM 10. | | |
| LEVEL OF HELP RECEIVED (FRIENDS OR RELATIVES)HOW MUCH HELP DOES THE PERSON RECEIVE FROM FRIENDS OR RELATIVES FOR THIS DISTRESS? | Can 0903 |  |
|  |  |  |
| **□□□□□□□□□□□** **□** NOT KNOWN  None Very high help  NONE e.g. No support.  VERY HIGH HELP e.g. Constant support and supervision. | | |
| LEVEL OF HELP RECEIVED (SERVICES)HOW MUCH HELP DOES THE PERSON RECEIVE FROM LOCAL SERVICES FOR THIS DISTRESS? | Can 0905 |  |
|  |  |  |
| **□□□□□□□□□□□** **□** NOT KNOWN  None Very high help  NONE e.g. No consultation.  VERY HIGH HELP e.g. 24-hour hospital care or crisis care. | | |
| **LEVEL OF HELP NEEDED (SERVICES)** HOW MUCH HELP DOES THE PERSON NEED FROM LOCAL SERVICES FOR THIS DISTRESS? | Can 0907 |  |
|  |  |  |
| **□□□□□□□□□□□** **□** NOT KNOWN  None Very high help  NONE e.g. No consultation needed.  VERY HIGH HELP e.g. 24-hour hospital care or crisis care. | | |

| APPROPRIATENESS OF CARE (SERVICES)DOES THE PERSON RECEIVE THE RIGHT TYPE OF HELP FOR THIS DISTRESS? | Can 0909 |  |
| --- | --- | --- |
|  |  |
| **QUANTITY**  **□□□□□□□□□□□** **□** NOT KNOWN  Not at all Entirely satisfied  satisfied | | |
| APPROPRIATENESS OF CARE (SERVICES)DOES THE PERSON RECEIVE THE RIGHT TYPE OF HELP FOR THIS DISTRESS? | Can 0911 |  |
|  |  |
| **QUALITY**  **□□□□□□□□□□□** **□** NOT KNOWN  Not at all Entirely satisfied  satisfied | | |
| USER’S PERSPECTIVE OF SERVICES REQUIRED | | |
| Thinking back about services received for your psychological distress, tell us in a few words what you most appreciated. (Write down respondent’s spontaneously given information)  In your experience, what could be improved? (Write down respondent’s spontaneously given information) | | |

| 10. SAFETY TO SELF | Assessments | |
| --- | --- | --- |
|  | *User* |  |
| IS THE PERSON A DANGER TO THEMSELVES?*Do you ever have thoughts of harming yourself, or actually harm yourself?**Do you put yourself in danger in other ways?* | Can 1001 |  |
|  |  |  |
| Please rate importance of current problem.  **□□□□□□□□□□□** **□** NOT KNOWN  No problem Very serious problem IF RATING IS *NO PROBLEM* OR *NOT KNOWN*, GO TO ITEM 11. | | |
| LEVEL OF HELP RECEIVED (FRIENDS OR RELATIVES)HOW MUCH HELP DOES THE PERSON RECEIVE FROM FRIENDS OR RELATIVES TO REDUCE THE RISK THAT THEY MIGHT HARM THEMSELF? | Can 1003 |  |
|  |  |  |
| **□□□□□□□□□□□** **□** NOT KNOWN  None Very high help  NONE e.g. No contact.  VERY HIGH HELP e.g. Friends or relatives in regular contact and are very likely to know and provide help if feeling unsafe. | | |
| **LEVEL OF HELP RECEIVED (SERVICES)** HOW MUCH HELP DOES THE PERSON RECEIVE FROM LOCAL SERVICES TO REDUCE THE RISK THAT THEY MIGHT HARM THEMSELF? | Can 1005 |  |
|  |  |  |
| **□□□□□□□□□□□** **□** NOT KNOWN  None Very high help  NONE e.g. Receives no supervision.  VERY HIGH HELP e.g. Daily supervision, in-patient care. | | |
| **LEVEL OF HELP NEEDED (SERVICES)** HOW MUCH HELP DOES THE PERSON NEED FROM LOCAL SERVICES TO REDUCE THE RISK THAT THEY MIGHT HARM THEMSELF? | Can 1007 |  |
|  |  |  |
| **□□□□□□□□□□□** **□** NOT KNOWN  None Very high help  NONE e.g. No supervision needed.  VERY HIGH HELP e.g. Daily supervision, inpatient care. | | |

| APPROPRIATENESS OF CARE (SERVICES)DOES THE PERSON RECEIVE THE RIGHT TYPE OF HELP TO REDUCE THE RISK THAT THEY MIGHT HARM THEMSELF? | Can 1009 |  |
| --- | --- | --- |
|  |  |
| **QUANTITY**  **□□□□□□□□□□□** **□** NOT KNOWN  Not at all Entirely satisfied  satisfied | | |
| APPROPRIATENESS OF CARE (SERVICES)DOES THE PERSON RECEIVE THE RIGHT TYPE OF HELP TO REDUCE THE RISK THAT THEY MIGHT HARM THEMSELF? | Can 1011 |  |
|  |  |
| **QUALITY**  **□□□□□□□□□□□** **□** NOT KNOWN  Not at all Entirely satisfied  satisfied | | |
| USER’S PERSPECTIVE OF SERVICES REQUIRED | | |
| Thinking back about services received in reducing the risk to harm yourself, tell us in a few words what you most appreciated. (Write down respondent’s spontaneously given information)  In your experience, what could be improved? (Write down respondent’s spontaneously given information) | | |

| 11. SAFETY TO OTHERS | Assessments | |
| --- | --- | --- |
|  | *User* |  |
| IS THE PERSON A CURRENT POTENTIAL RISK TO OTHER PEOPLE’S SAFETY?*Do you think you could be a danger to other people’s safety?* *Do you ever lose your temper and hit someone?* | Can 1101 |  |
|  |  |  |
| Please rate importance of current problem.  **□□□□□□□□□□□** **□** NOT KNOWN  No problem Very serious problem IF RATING IS *NO PROBLEM* OR *NOT KNOWN*, GO TO ITEM 12. | | |
| LEVEL OF HELP RECEIVED (FRIENDS OR RELATIVES)HOW MUCH HELP DOES THE PERSON RECEIVE FROM FRIENDS OR RELATIVES TO REDUCE THE RISK THAT THEY MIGHT HARM SOMEONE ELSE? | Can 1103 |  |
|  |  |  |
| **□□□□□□□□□□□** **□** NOT KNOWN  None Very high help  NONE e.g. Receives no help.  VERY HIGH HELP e.g. Almost constant help with persistently threatening behaviour. | | |
| LEVEL OF HELP RECEIVED (SERVICES)HOW MUCH HELP DOES THE PERSON RECEIVE FROM LOCAL SERVICES TO REDUCE THE RISK THAT THEY MIGHT HARM SOMEONE ELSE? | Can 1105 |  |
|  |  |  |
| **□□□□□□□□□□□** **□** NOT KNOWN  None Very high help  NONE e.g. Receives no supervision.  VERY HIGH HELP e.g. Constant supervision. Anger-management program. | | |
| LEVEL OF HELP NEEDED (SERVICES)HOW MUCH HELP DOES THE PERSON NEED FROM LOCAL SERVICES TO REDUCE THE RISK THAT THEY MIGHT HARM SOMEONE ELSE? | Can 1107 |  |
|  |  |  |
| **□□□□□□□□□□□** **□** NOT KNOWN  None Very high help  NONE e.g. No supervision needed.  VERY HIGH HELP e.g. Constant supervision. Anger management program. | | |

| APPROPRIATENESS OF CARE (SERVICES)DOES THE PERSON RECEIVE THE RIGHT TYPE OF HELP TO REDUCE THE RISK THAT THEY MIGHT HARM SOMEONE ELSE? | Can 1109 |  |
| --- | --- | --- |
|  |  |
| **QUANTITY**  **□□□□□□□□□□□** **□** NOT KNOWN  Not at all Entirely satisfied  satisfied | | |
| APPROPRIATENESS OF CARE (SERVICES)DOES THE PERSON RECEIVE THE RIGHT TYPE OF HELP TO REDUCE THE RISK THAT THEY MIGHT HARM SOMEONE ELSE? | Can 1111 |  |
|  |  |
| **QUALITY**  **□□□□□□□□□□□** **□** NOT KNOWN  Not at all Entirely satisfied  satisfied | | |
| USER’S PERSPECTIVE OF SERVICES REQUIRED | | |
| Thinking back about services received in reducing the risk to harm someone else, tell us in a few words what you most appreciated. (Write down respondent’s spontaneously given information)  In your experience, what could be improved? (Write down respondent’s spontaneously given information) | | |

| 12. ALCOHOL | Assessments | |
| --- | --- | --- |
|  | *User* |  |
| DOES THE PERSON DRINK EXCESSIVELY, OR HAVE A PROBLEM CONTROLLING THEIR DRINKING?*Does drinking cause you any problems?**Do you wish you could cut down your drinking?* | Can 1201 |  |
|  |  |  |
| Please rate importance of current problem.  **□□□□□□□□□□□** **□** NOT KNOWN  No problem Very serious problem IF RATING IS *NO PROBLEM* OR *NOT KNOWN*, GO TO ITEM 13. | | |
| LEVEL OF HELP RECEIVED (FRIENDS OR RELATIVES) **HOW MUCH HELP DOES THE PERSON RECEIVE FROM FRIENDS OR RELATIVES FOR THEIR DRINKING?** | Can 1203 |  |
|  |  |  |
| **□□□□□□□□□□□** **□** NOT KNOWN  None Very high help  NONE e.g. Receives no help.  VERY HIGH HELP e.g. Daily monitoring of alcohol. | | |
| LEVEL OF HELP RECEIVED (SERVICES)HOW MUCH HELP DOES THE PERSON RECEIVE FROM LOCAL SERVICES FOR THEIR DRINKING? | Can 1205 |  |
|  |  |  |
| **□□□□□□□□□□□** **□** NOT KNOWN  None Very high help  NONE e.g. Receives no help  VERY HIGH HELP e.g. Attends alcohol clinic, supervised withdrawal program. | | |
| **LEVEL OF HELP NEEDED (SERVICES)** HOW MUCH HELP DOES THE PERSON NEED FROM LOCAL SERVICES FOR THEIR DRINKING? | Can 1207 |  |
|  |  |  |
| **□□□□□□□□□□□** **□** NOT KNOWN  None Very high help  NONE e.g. Help not needed.  VERY HIGH HELP e.g. Attends alcohol clinic, supervised withdrawal program. | | |

| APPROPRIATENESS OF CARE (SERVICES)DOES THE PERSON RECEIVE THE RIGHT TYPE OF HELP FOR THEIR DRINKING? | Can 1209 |  |
| --- | --- | --- |
|  |  |
| **QUANTITY**  **□□□□□□□□□□□** **□** NOT KNOWN  Not at all Entirely satisfied  satisfied | | |
| APPROPRIATENESS OF CARE (SERVICES)DOES THE PERSON RECEIVE THE RIGHT TYPE OF HELP FOR THEIR DRINKING? | Can 1211 |  |
|  |  |
| **QUALITY**  **□□□□□□□□□□□** **□** NOT KNOWN  Not at all Entirely satisfied  satisfied | | |
| USER’S PERSPECTIVE OF SERVICES REQUIRED | | |
| Thinking back about services received for your drinking, tell us in a few words what you most appreciated. (Write down respondent’s spontaneously given information)  In your experience, what could be improved? (Write down respondent’s spontaneously given information) | | |

| 13. DRUGS | Assessments | |
| --- | --- | --- |
|  | *User* |  |
| DOES THE PERSON HAVE PROBLEMS WITH DRUG ABUSE?*Do you take any drugs that aren’t prescribed?**Are there any drugs you would find hard to stop taking?* | Can 1301 |  |
|  |  |  |
| Please rate importance of current problem.  **□□□□□□□□□□□** **□** NOT KNOWN  No problem Very serious problem IF RATING IS *NO PROBLEM* OR *NOT KNOWN*, GO TO ITEM 14. | | |
| LEVEL OF HELP RECEIVED (FRIENDS OR RELATIVES)HOW MUCH HELP DOES THE PERSON RECEIVE FROM FRIENDS OR RELATIVES FOR THEIR DRUG ABUSE? | Can 1303 |  |
|  |  |  |
| **□□□□□□□□□□□** **□** NOT KNOWN  None Very high help  NONE e.g. Receives no help.  VERY HIGH HELP e.g. Supervision, liaison with other agencies. | | |
| LEVEL OF HELP RECEIVED (SERVICES)HOW MUCH HELP DOES THE PERSON RECEIVE FROM LOCAL SERVICES FOR THEIR DRUG ABUSE? | Can 1305 |  |
|  |  |  |
| **□□□□□□□□□□□** **□** NOT KNOWN  None Very high help  NONE e.g. Receives no supervision.  VERY HIGH HELP e.g. Supervised withdrawal program, in-patient care. | | |
| **LEVEL OF HELP NEEDED (SERVICES)** HOW MUCH HELP DOES THE PERSON NEED FROM LOCAL SERVICES FOR THEIR DRUG ABUSE? | Can 1307 |  |
|  |  |  |
| **□□□□□□□□□□□** **□** NOT KNOWN  None Very high help  NONE e.g. No supervision needed.  VERY HIGH HELP e.g. Supervised withdrawal program, in-patient care. . | | |

| APPROPRIATENESS OF CARE (SERVICES)DOES THE PERSON RECEIVE THE RIGHT TYPE OF HELP FOR THEIR DRUG ABUSE? | Can 1309 |  |
| --- | --- | --- |
|  |  |
| **QUANTITY**  **□□□□□□□□□□□** **□** NOT KNOWN  Not at all Entirely satisfied  satisfied | | |
| APPROPRIATENESS OF CARE (SERVICES)DOES THE PERSON RECEIVE THE RIGHT TYPE OF HELP FOR THEIR DRUG ABUSE? | Can 1311 |  |
|  |  |
| **QUALITY**  **□□□□□□□□□□□** **□** NOT KNOWN  Not at all Entirely satisfied  satisfied | | |
| USER’S PERSPECTIVE OF SERVICES REQUIRED | | |
| Thinking back about services received for your drug abuse, tell us in a few words what you most appreciated. (Write down respondent’s spontaneously given information)  In your experience, what could be improved? (Write down respondent’s spontaneously given information) | | |

| 14. COMPANY | Assessments | |
| --- | --- | --- |
|  | *User* |  |
| DOES THE PERSON NEED HELP WITH SOCIAL CONTACT?*Are you happy with your social life?**Do you feel alone? Do you wish you had more contact with others?* | Can 1401 |  |
|  |  |  |
| Please rate importance of current problem.  **□□□□□□□□□□□** **□** NOT KNOWN  No problem Very serious problem IF RATING IS *NO PROBLEM* OR *NOT KNOWN*, GO TO ITEM 15. | | |
| LEVEL OF HELP RECEIVED (FRIENDS OR RELATIVES)HOW MUCH HELP DOES THE PERSON RECEIVE FROM FRIENDS OR RELATIVES WITH SOCIAL CONTACT? | Can 1403 |  |
|  |  |  |
| **□□□□□□□□□□□** **□** NOT KNOWN  None Very high help  NONE e.g. Receives no help.  VERY HIGH HELP e.g. Social contact at least four times a week. | | |
| LEVEL OF HELP RECEIVED (SERVICES)HOW MUCH HELP DOES THE PERSON RECEIVE FROM LOCAL SERVICES IN ORGANIZING SOCIAL CONTACT? | Can 1405 |  |
|  |  |  |
| **□□□□□□□□□□□** **□** NOT KNOWN  None Very high help  NONE e.g. Receives no help.  VERY HIGH HELP e.g. Attends day centre 4 or more times a week. | | |
| **LEVEL OF HELP NEEDED (SERVICES)** HOW MUCH HELP DOES THE PERSON NEED FROM LOCAL SERVICES IN ORGANIZING SOCIAL CONTACT? | Can 1407 |  |
|  |  |  |
| **□□□□□□□□□□□** **□** NOT KNOWN  None Very high help  NONE e.g. No help needed.  VERY HIGH HELP e.g. Attends day centre 4 or more times a week. | | |

| APPROPRIATENESS OF CARE (SERVICES)DOES THE PERSON RECEIVE THE RIGHT TYPE OF HELP FOR IN ORGANIZING SOCIAL CONTACT? | Can 1409 |  |
| --- | --- | --- |
|  |  |
| **QUANTITY**  **□□□□□□□□□□□** **□** NOT KNOWN  Not at all Entirely satisfied  satisfied | | |
| APPROPRIATENESS OF CARE (SERVICES)DOES THE PERSON RECEIVE THE RIGHT TYPE OF HELP FOR IN ORGANIZING SOCIAL CONTACT? | Can 1411 |  |
|  |  |
| **QUALITY**  **□□□□□□□□□□□** **□** NOT KNOWN  Not at all Entirely satisfied  satisfied | | |
| USER’S PERSPECTIVE OF SERVICES REQUIRED | | |
| Thinking back about services received in organizing social contact, tell us in a few words what you most appreciated. (Write down respondent’s spontaneously given information)  In your experience, what could be improved? (Write down respondent’s spontaneously given information) | | |

| 15. INTIMATE RELATIONSHIPS | Assessments | | |
| --- | --- | --- | --- |
|  | *User* |  | |
| DOES THE PERSON HAVE ANY DIFFICULTY IN FINDING A PARTNER OR IN MAINTAINING A CLOSE RELATIONSHIP?*Do you have a partner?**Do you have problems in your partnership / marriage?* | Can 1501 |  | |
|  |  |  |  |
| Please rate importance of current problem.  **□□□□□□□□□□□** **□** NOT KNOWN  No problem Very serious problem IF RATING IS *NO PROBLEM* OR *NOT KNOWN*, GO TO ITEM 16. | | | |
| LEVEL OF HELP RECEIVED (FRIENDS OR RELATIVES)HOW MUCH HELP DOES THE PERSON RECEIVE FROM FRIENDS OR RELATIVES WITH FORMING AND MAINTAINING RELATIONSHIPS? | Can 1503 |  | |
|  |  |  |  |
| **□□□□□□□□□□□** **□** NOT KNOWN  None Very high help  NONE e.g. Receives no support.  VERY HIGH HELP e.g. Intensive talks and support in coping with feelings. | | | |
| LEVEL OF HELP RECEIVED (SERVICES)HOW MUCH HELP DOES THE PERSON RECEIVE FROM LOCAL SERVICES WITH FORMING AND MAINTAINING RELATIONSHIPS? | Can 1505 |  | |
|  |  |  |  |
| **□□□□□□□□□□□** **□** NOT KNOWN  None Very high help  NONE e.g. Receives no support.  VERY HIGH HELP e.g. Couple therapy, social skills training. | | |  |
| **LEVEL OF HELP NEEDED (SERVICES)** HOW MUCH HELP DOES THE PERSON NEED FROM LOCAL SERVICES WITH FORMING AND MAINTAINING RELATIONSHIPS? | Can 1507 |  | |
|  |  |  |  |
| **□□□□□□□□□□□** **□** NOT KNOWN  None Very high help  NONE e.g. No support needed.  VERY HIGH HELP e.g. Couple therapy, social skills training. | | | |

| APPROPRIATENESS OF CARE (SERVICES)DOES THE PERSON RECEIVE THE RIGHT TYPE OF HELP WITH FORMING AND MAINTAINING RELATIONSHIPS? | Can 1509 |  |
| --- | --- | --- |
|  |  |
| **QUANTITY**  **□□□□□□□□□□□** **□** NOT KNOWN  Not at all Entirely satisfied  satisfied | | |
| APPROPRIATENESS OF CARE (SERVICES)DOES THE PERSON RECEIVE THE RIGHT TYPE OF HELP WITH FORMING AND MAINTAINING RELATIONSHIPS? | Can 1511 |  |
|  |  |
| **QUALITY**  **□□□□□□□□□□□** **□** NOT KNOWN  Not at all Entirely satisfied  satisfied | | |
| USER’S PERSPECTIVE OF SERVICES REQUIRED | | |
| Thinking back about services received in forming and maintaining relationships, tell us in a few words what you most appreciated. (Write down respondent’s spontaneously given information)  In your experience, what could be improved? (Write down respondent’s spontaneously given information) | | |

| 16. SEXUAL EXPRESSION | Assessments | |
| --- | --- | --- |
|  | *User* |  |
| DOES THE PERSON HAVE PROBLEMS WITH THEIR SEX LIFE?*How is your sex life?* | Can 1601 |  |
|  |  |  |
| Please rate importance of current problem.  **□□□□□□□□□□□** **□** NOT KNOWN  No problem Very serious problem IF RATING IS *NO PROBLEM* OR *NOT KNOWN*, GO TO ITEM 17. | | |
| LEVEL OF HELP RECEIVED (FRIENDS OR RELATIVES)HOW MUCH HELP DOES THE PERSON RECEIVE FROM FRIENDS OR RELATIVES WITH PROBLEMS IN THEIR SEX LIFE? | Can 1603 |  |
|  |  |  |
| **□□□□□□□□□□□** **□** NOT KNOWN  None Very high help  NONE e.g. Receives no support.  VERY HIGH HELP e.g. Consistent accessibility to talk about the problem. | | |
| LEVEL OF HELP RECEIVED (SERVICES)HOW MUCH HELP DOES THE PERSON RECEIVE FROM LOCAL SERVICES FOR PROBLEMS IN THEIR SEX LIFE? | Can 1605 |  |
|  |  |  |
| **□□□□□□□□□□□** **□** NOT KNOWN  None Very high help  NONE e.g. Receives no support.  VERY HIGH HELP e.g. Sexual therapy. | | |
| **LEVEL OF HELP NEEDED (SERVICES)** HOW MUCH HELP DOES THE PERSON NEED FROM LOCAL SERVICES FOR PROBLEMS IN THEIR SEX LIFE? | Can 1607 |  |
|  |  |  |
| **□□□□□□□□□□□** **□** NOT KNOWN  None Very high help  NONE e.g. No help needed.  VERY HIGH HELP e.g. Sexual therapy. | | |

| APPROPRIATENESS OF CARE (SERVICES)DOES THE PERSON RECEIVE THE RIGHT TYPE OF HELP FOR PROBLEMS IN THEIR SEX LIFE? | Can 1609 |  |
| --- | --- | --- |
|  |  |
| **QUANTITY**  **□□□□□□□□□□□** **□** NOT KNOWN  Not at all Entirely satisfied  satisfied | | |
| APPROPRIATENESS OF CARE (SERVICES)DOES THE PERSON RECEIVE THE RIGHT TYPE OF HELP FOR PROBLEMS IN THEIR SEX LIFE? | Can 1611 |  |
|  |  |
| **QUALITY**  **□□□□□□□□□□□** **□** NOT KNOWN  Not at all Entirely satisfied  satisfied | | |
| USER’S PERSPECTIVE OF SERVICES REQUIRED | | |
| Thinking back about services received for problems in your sex life, tell us in a few words what you most appreciated. (Write down respondent’s spontaneously given information)  In your experience, what could be improved? (Write down respondent’s spontaneously given information) | | |

| 17. CHILDCAREIF THE PERSON HAS NO CHILDREN UNDER 18 YEARS OLD, GO TO ITEM 18. | Assessments | |
| --- | --- | --- |
|  | *User* |  |
| DOES THE PERSON HAVE DIFFICULTY LOOKING AFTER THEIR CHILDREN?*Do you have any children under 18?**Do you have any difficulty in looking after them?* | Can 1701 |  |
|  |  |  |
| Please rate importance of current problem.  **□□□□□□□□□□□** **□** NOT KNOWN  No problem Very serious problem IF RATING IS *NO PROBLEM* OR *NOT KNOWN*, GO TO ITEM 18. | | |
| LEVEL OF HELP RECEIVED (FRIENDS OR RELATIVES)HOW MUCH HELP DOES THE PERSON RECEIVE FROM FRIENDS OR RELATIVES WITH LOOKING AFTER THEIR CHILDREN? | Can 1703 |  |
|  |  |  |
| **□□□□□□□□□□□** **□** NOT KNOWN  None Very high help  NONE e.g. Receives no help.  VERY HIGH HELP e.g. Children living with friends or relatives. | | |
| LEVEL OF HELP RECEIVED (SERVICES)HOW MUCH HELP DOES THE PERSON RECEIVE FROM LOCAL SERVICES WITH LOOKING AFTER THEIR CHILDREN? | Can 1705 |  |
|  |  |  |
| **□□□□□□□□□□□** **□** NOT KNOWN  None Very high help  NONE e.g. Receives no help.  VERY HIGH HELP e.g. Children in foster home, or in care. | | |
| **LEVEL OF HELP NEEDED (SERVICES)** HOW MUCH HELP DOES THE PERSON NEED FROM LOCAL SERVICES WITH LOOKING AFTER THEIR CHILDREN? | Can 1707 |  |
|  |  |  |
| **□□□□□□□□□□□** **□** NOT KNOWN  None Very high help  NONE e.g. No help needed.  VERY HIGH HELP e.g. Children in foster home, or in care. | | |

| APPROPRIATENESS OF CARE (SERVICES)DOES THE PERSON RECEIVE THE RIGHT TYPE OF HELP FOR LOOKING AFTER THEIR CHILDREN? | Can 1709 |  |
| --- | --- | --- |
|  |  |
| **QUANTITY**  **□□□□□□□□□□□** **□** NOT KNOWN  Not at all Entirely satisfied  satisfied | | |
| APPROPRIATENESS OF CARE (SERVICES)DOES THE PERSON RECEIVE THE RIGHT TYPE OF HELP FOR LOOKING AFTER THEIR CHILDREN? | Can 1711 |  |
|  |  |
| **QUALITY**  **□□□□□□□□□□□** **□** NOT KNOWN  Not at all Entirely satisfied  satisfied | | |
| USER’S PERSPECTIVE OF SERVICES REQUIRED | | |
| Thinking back about services received in looking after your children, tell us in a few words what you most appreciated. (Write down respondent’s spontaneously given information)  In your experience, what could be improved? (Write down respondent’s spontaneously given information) | | |

| 18. BASIC EDUCATION | Assessments | |
| --- | --- | --- |
|  | *User* |  |
| DOES THE PERSON LACK BASIC SKILLS IN NUMERACY AND LITERACY?*Do you have difficulty in reading, writing or understanding English?**Can you count your change in a shop?* | Can 1801 |  |
|  |  |  |
| Please rate importance of current problem.  **□□□□□□□□□□□** **□** NOT KNOWN  No problem Very serious problem IF RATING IS *NO PROBLEM* OR *NOT KNOWN*, GO TO ITEM 19. | | |
| LEVEL OF HELP RECEIVED (FRIENDS OR RELATIVES)HOW MUCH HELP DOES THE PERSON RECEIVE FROM FRIENDS OR RELATIVES WITH NUMERACY AND LITERACY? | Can 1803 |  |
|  |  |  |
| **□□□□□□□□□□□** **□** NOT KNOWN  None Very high help  NONE e.g. Receives no help.  VERY HIGH HELP e.g. Teaches the person to read. | | |
| LEVEL OF HELP RECEIVED (SERVICES)HOW MUCH HELP DOES THE PERSON RECEIVE FROM LOCAL SERVICES WITHNUMERACY AND LITERACY? | Can 1805 |  |
|  |  |  |
| **□□□□□□□□□□□** **□** NOT KNOWN  None Very high help  NONE e.g. Receives no help.  VERY HIGH HELP e.g. Attending adult education. | | |
| LEVEL OF HELP NEEDED (SERVICES)HOW MUCH HELP DOES THE PERSON NEED FROM LOCAL SERVICES WITH NUMERACY AND LITERACY? | Can 1807 |  |
|  |  |  |
| **□□□□□□□□□□□** **□** NOT KNOWN  None Very high help  NONE e.g. No help needed.  VERY HIGH HELP e.g. Attending adult education. | | |

| APPROPRIATENESS OF CARE (SERVICES)DOES THE PERSON RECEIVE THE RIGHT TYPE OF HELP WITH NUMERACY AND LITERACY? | Can 1809 |  |
| --- | --- | --- |
|  |  |
| **QUANTITY**  **□□□□□□□□□□□** **□** NOT KNOWN  Not at all Entirely satisfied  satisfied | | |
| APPROPRIATENESS OF CARE (SERVICES)DOES THE PERSON RECEIVE THE RIGHT TYPE OF HELP WITH NUMERACY AND LITERACY? | Can 1811 |  |
|  |  |
| **QUALITY**  **□□□□□□□□□□□** **□** NOT KNOWN  Not at all Entirely satisfied  satisfied | | |
| USER’S PERSPECTIVE OF SERVICES REQUIRED | | |
| Thinking back about services received in numeracy and literacy, tell us in a few words what you most appreciated. (Write down respondent’s spontaneously given information)  In your experience, what could be improved? (Write down respondent’s spontaneously given information) | | |

| 19. TELEPHONE | Assessments | |
| --- | --- | --- |
|  | *User* |  |
| DOES THE PERSON HAVE DIFFICULTY IN GETTING ACCESS TO OR USING A TELEPHONE?*Do you know how to use a telephone?**Is it easy to find one that you can use?* | Can 1901 |  |
|  |  |  |
| Please rate importance of current problem.  **□□□□□□□□□□□** **□** NOT KNOWN  No problem Very serious problem IF RATING IS *NO PROBLEM* OR *NOT KNOWN*, GO TO ITEM 20. | | |
| LEVEL OF HELP RECEIVED (FRIENDS OR RELATIVES) **HOW MUCH HELP DOES THE PERSON RECEIVE FROM FRIENDS OR RELATIVES TO MAKE TELEPHONE CALLS?** | Can 1903 |  |
|  |  |  |
| **□□□□□□□□□□□** **□** NOT KNOWN  None Very high help  NONE e.g. Receives no help.  VERY HIGH HELP e.g. Help available whenever wanted. | | |
| LEVEL OF HELP RECEIVED (SERVICES)HOW MUCH HELP DOES THE PERSON RECEIVE FROM LOCAL SERVICES TO MAKE TELEPHONE CALLS? | Can 1905 |  |
|  |  |  |
| **□□□□□□□□□□□** **□** NOT KNOWN  None Very high help  NONE e.g. Receives no help.  VERY HIGH HELP e.g. Arranges to have telephone fitted in home. | | |
| LEVEL OF HELP NEEDED (SERVICES)HOW MUCH HELP DOES THE PERSON NEED FROM LOCAL SERVICES TO MAKE TELEPHONE CALLS? | Can 1907 |  |
|  |  |  |
| **□□□□□□□□□□□** **□** NOT KNOWN  None Very high help  NONE e.g. No help needed.  VERY HIGH HELP e.g. Arranges to have telephone fitted in home. | | |

| APPROPRIATENESS OF CARE (SERVICES)DOES THE PERSON RECEIVE THE RIGHT TYPE OF HELP TO MAKE TELEPHONE CALLS? | Can 1909 |  |
| --- | --- | --- |
|  |  |
| **QUANTITY**  **□□□□□□□□□□□** **□** NOT KNOWN  Not at all Entirely satisfied  satisfied | | |
| APPROPRIATENESS OF CARE (SERVICES)DOES THE PERSON RECEIVE THE RIGHT TYPE OF HELP TO MAKE TELEPHONE CALLS? | Can 1911 |  |
|  |  |
| **QUALITY**  **□□□□□□□□□□□** **□** NOT KNOWN  Not at all Entirely satisfied  satisfied | | |
| USER’S PERSPECTIVE OF SERVICES REQUIRED | | |
| Thinking back about services received in making telephone calls, tell us in a few words what you most appreciated. (Write down respondent’s spontaneously given information)  In your experience, what could be improved? (Write down respondent’s spontaneously given information) | | |

| 20. TRANSPORT | Assessments | |
| --- | --- | --- |
|  | *User* |  |
| DOES THE PERSON HAVE ANY PROBLEMS USING PUBLIC TRANSPORT?*How do you find using the bus, subway or train?**Do you get a free bus pass?* | Can 2001 |  |
|  |  |  |
| Please rate importance of current problem.  **□□□□□□□□□□□** **□** NOT KNOWN  No problem Very serious problem IF RATING IS *NO PROBLEM* OR *NOT KNOWN*, GO TO ITEM 21. | | |
| LEVEL OF HELP RECEIVED (FRIENDS OR RELATIVES)HOW MUCH HELP DOES THE PERSON RECEIVE FROM FRIENDS OR RELATIVES WITH TRAVELLING? | Can 2003 |  |
|  |  |  |
| **□□□□□□□□□□□** **□** NOT KNOWN  None Very high help  NONE e.g. Not accompanied.  VERY HIGH HELP e.g. Provides transport to all appointments. | | |
| LEVEL OF HELP RECEIVED (SERVICES)HOW MUCH HELP DOES THE PERSON RECEIVE FROM LOCAL SERVICES WITH TRAVELLING? | Can 2005 |  |
|  |  |  |
| **□□□□□□□□□□□** **□** NOT KNOWN  None Very high help  NONE e.g. Receives no help.  VERY HIGH HELP e.g. Transport to appointments by ambulance. | | |
| **LEVEL OF HELP NEEDED (SERVICES)** HOW MUCH HELP DOES THE PERSON NEED FROM LOCAL SERVICES WITH TRAVELLING? | Can 2007 |  |
|  |  |  |
| **□□□□□□□□□□□** **□** NOT KNOWN  None Very high help  NONE e.g. Help not needed.  VERY HIGH HELP e.g. Transport to appointments by ambulance. | | |

| APPROPRIATENESS OF CARE (SERVICES)DOES THE PERSON RECEIVE THE RIGHT TYPE OF HELP WITH TRAVELLING? | Can 2009 |  |
| --- | --- | --- |
|  |  |
| **QUANTITY**  **□□□□□□□□□□□** **□** NOT KNOWN  Not at all Entirely satisfied  satisfied | | |
| APPROPRIATENESS OF CARE (SERVICES)DOES THE PERSON RECEIVE THE RIGHT TYPE OF HELP WITH TRAVELLING? | Can 2011 |  |
|  |  |
| **QUALITY**  **□□□□□□□□□□□** **□** NOT KNOWN  Not at all Entirely satisfied  satisfied | | |
| USER’S PERSPECTIVE OF SERVICES REQUIRED | | |
| Thinking back about services received in using public transport, tell us in a few words what you most appreciated. (Write down respondent’s spontaneously given information)  In your experience, what could be improved? (Write down respondent’s spontaneously given information) | | |

| 21. MONEY | Assessments | |
| --- | --- | --- |
|  | *User* |  |
| DOES THE PERSON HAVE PROBLEMS BUDGETING THEIR MONEY?*How do you find budgeting your money?**Do you manage to pay your bills?* | Can 2101 |  |
|  |  |  |
| Please rate importance of current problem.  **□□□□□□□□□□□** **□** NOT KNOWN  No problem Very serious problem IF RATING IS *NO PROBLEM* OR *NOT KNOWN*, GO TO ITEM 22. | | |
| LEVEL OF HELP RECEIVED (FRIENDS OR RELATIVES) **HOW MUCH HELP DOES THE PERSON RECEIVE FROM FRIENDS OR RELATIVES IN MANAGING THEIR MONEY?** | Can 2103 |  |
|  |  |  |
| **□□□□□□□□□□□** **□** NOT KNOWN  None Very high help  NONE e.g. Receives no help.  VERY HIGH HELP e.g. Complete control or finances. | | |
| LEVEL OF HELP RECEIVED (SERVICES)HOW MUCH HELP DOES THE PERSON RECEIVE FROM LOCAL SERVICES IN MANAGING THEIR MONEY? | Can 2105 |  |
|  |  |  |
| **□□□□□□□□□□□** **□** NOT KNOWN  None Very high help  NONE e.g. Receives no help.  VERY HIGH HELP e.g. Daily handouts of cash. | | |
| LEVEL OF HELP NEEDED (SERVICES)HOW MUCH HELP DOES THE PERSON NEED FROM LOCAL SERVICES IN MANAGING THEIR MONEY? | Can 2107 |  |
|  |  |  |
| **□□□□□□□□□□□** **□** NOT KNOWN  None Very high help  NONE e.g. No help needed.  VERY HIGH HELP e.g. Daily handouts of cash. | | |

| APPROPRIATENESS OF CARE (SERVICES)DOES THE PERSON RECEIVE THE RIGHT TYPE OF HELP IN MANAGING THEIR MONEY? | Can 2109 |  |
| --- | --- | --- |
|  |  |
| **QUANTITY**  **□□□□□□□□□□□** **□** NOT KNOWN  Not at all Entirely satisfied  satisfied | | |
| APPROPRIATENESS OF CARE (SERVICES)DOES THE PERSON RECEIVE THE RIGHT TYPE OF HELP IN MANAGING THEIR MONEY? | Can 2111 |  |
|  |  |
| **QUALITY**  **□□□□□□□□□□□** **□** NOT KNOWN  Not at all Entirely satisfied  satisfied | | |
| USER’S PERSPECTIVE OF SERVICES REQUIRED | | |
| Thinking back about services received in managing your money, tell us in a few words what you most appreciated. (Write down respondent’s spontaneously given information)  In your experience, what could be improved? (Write down respondent’s spontaneously given information) | | |

| 22. SOCIAL BENEFITS | Assessments | |
| --- | --- | --- |
|  | *User* |  |
| IS THE PERSON RECEIVING ALL THE FINANCIAL BENEFITS FROM THE GOVERNMENT THAT THEY ARE ENTITLED TO RECEIVE?*Are you sure that you are getting all the money you are entitled to receive?* | Can 2201 |  |
|  |  |  |
| Please rate importance of current problem.  **□□□□□□□□□□□** **□** NOT KNOWN  No problem Very serious problem IF RATING IS *NO PROBLEM* OR *NOT KNOWN*, GO TO ITEM 23. | | |
| LEVEL OF HELP RECEIVED (FRIENDS OR RELATIVES)HOW MUCH HELP DOES THE PERSON RECEIVE FROM FRIENDS OR RELATIVES IN OBTAINING FULL FINANCIAL ENTITLEMENT FROM THE GOVERNMENT? | Can 2203 |  |
|  |  |  |
| **□□□□□□□□□□□** **□** NOT KNOWN  None Very high help  NONE e.g. Receives no help.  VERY HIGH HELP e.g. Has made enquiries about full entitlement. | | |
| LEVEL OF HELP RECEIVED (SERVICES)HOW MUCH HELP DOES THE PERSON RECEIVE FROM LOCAL SERVICES IN OBTAINING FULL FINANCIAL ENTITLEMENT FROM THE GOVERNMENT? | Can 2205 |  |
|  |  |  |
| **□□□□□□□□□□□** **□** NOT KNOWN  None Very high help  NONE e.g. Receives no help.  VERY HIGH HELP e.g. Comprehensive evaluation of current government entitlement. | | |
| **LEVEL OF HELP NEEDED (SERVICES)** HOW MUCH HELP DOES THE PERSON NEED FROM LOCAL SERVICES IN OBTAINING FULL FINANCIAL ENTITLEMENT FROM THE GOVERNMENT? | Can 2207 |  |
|  |  |  |
| **□□□□□□□□□□□** **□** NOT KNOWN  None Very high help  NONE e.g. No help needed.  VERY HIGH HELP e.g. Comprehensive evaluation of current government entitlement. | | |

| APPROPRIATENESS OF CARE (SERVICES)DOES THE PERSON RECEIVE THE RIGHT TYPE OF HELP IN OBTAINING FULL FINANCIAL ENTITLEMENT FROM THE GOVERNMENT? | Can 2209 |  |
| --- | --- | --- |
|  |  |
| **QUANTITY**  **□□□□□□□□□□□** **□** NOT KNOWN  Not at all Entirely satisfied  satisfied | | |
| APPROPRIATENESS OF CARE (SERVICES)DOES THE PERSON RECEIVE THE RIGHT TYPE OF HELP IN OBTAINING FULL FINANCIAL ENTITLEMENT FROM THE GOVERNMENT? | Can 2211 |  |
|  |  |
| **QUALITY**  **□□□□□□□□□□□** **□** NOT KNOWN  Not at all Entirely satisfied  satisfied | | |
| USER’S PERSPECTIVE OF SERVICES REQUIRED | | |
| Thinking back about services received in obtaining governmental financial benefits from the government, tell us in a few words what you most appreciated. (Write down respondent’s spontaneously given information)  In your experience, what could be improved? (Write down respondent’s spontaneously given information) | | |

| 23. WORK | Assessments | |
| --- | --- | --- |
|  | *User* |  |
| DOES THE PERSON HAVE PROBLEMS FINDING OR KEEPING EMPLOYMENT?*Are you currently working?* | Can 2301 |  |
|  |  |  |
| Please rate importance of current problem.  **□□□□□□□□□□□** **□** NOT KNOWN  No problem Very serious problem IF RATING IS *NO PROBLEM* OR *NOT KNOWN*, GO TO ITEM 24. | | |
| LEVEL OF HELP RECEIVED (FRIENDS OR RELATIVES)HOW MUCH HELP DOES THE PERSON RECEIVE FROM FRIENDS OR RELATIVES IN FINDING AND KEEPING EMPLOYMENT? | Can 2303 |  |
|  |  |  |
| **□□□□□□□□□□□** **□** NOT KNOWN  None Very high help  NONE e.g. Receives no help.  VERY HIGH HELP e.g. Accompanies in consulting return-to-workforce services. | | |
| LEVEL OF HELP RECEIVED (SERVICES)HOW MUCH HELP DOES THE PERSON RECEIVE FROM LOCAL SERVICES WITH FINDING AND KEEPING EMPLOYMENT? | Can 2305 |  |
|  |  |  |
| **□□□□□□□□□□□** **□** NOT KNOWN  None Very high help  NONE e.g. Receives no information.  VERY HIGH HELP e.g. Intensive support in working environment. | | |
| **LEVEL OF HELP NEEDED (SERVICES)** HOW MUCH HELP DOES THE PERSON NEED FROM LOCAL SERVICES WITH FINDING AND KEEPING EMPLOYMENT? | Can 2307 |  |
|  |  |  |
| **□□□□□□□□□□□** **□** NOT KNOWN  None Very high help  NONE e.g. No information needed.  MODERATE HELP e.g. Training.  VERY HIGH HELP e.g. Intensive support in working environment. | | |

| APPROPRIATENESS OF CARE (SERVICES)DOES THE PERSON RECEIVE THE RIGHT TYPE OF HELP WITH FINDING AND KEEPING EMPLOYMENT? | Can 2309 |  |
| --- | --- | --- |
|  |  |
| **QUANTITY**  **□□□□□□□□□□□** **□** NOT KNOWN  Not at all Entirely satisfied  satisfied | | |
| APPROPRIATENESS OF CARE (SERVICES)DOES THE PERSON RECEIVE THE RIGHT TYPE OF HELP WITH FINDING AND KEEPING EMPLOYMENT? | Can 2311 |  |
|  |  |
| **QUALITY**  **□□□□□□□□□□□** **□** NOT KNOWN  Not at all Entirely satisfied  satisfied | | |
| USER’S PERSPECTIVE OF SERVICES REQUIRED | | |
| Thinking back about services received in finding and keeping employment, tell us in a few words what you most appreciated. (Write down respondent’s spontaneously given information)  In your experience, what could be improved? (Write down respondent’s spontaneously given information) | | |

| 24. ADAPTATION TO DAILY STRESS | Assessments | |
| --- | --- | --- |
|  | *User* |  |
| DOES THE PERSON HAVE DIFFICULTY MANAGING THEIR DAILY STRESS?*Are you able to face unforeseen elements of daily life?* | Can 2401 |  |
|  |  |  |
| Please rate importance of current problem.  **□□□□□□□□□□□** **□** NOT KNOWN  No problem Very serious problem IF RATING IS *NO PROBLEM* OR *NOT KNOWN*, GO TO ITEM 25. | | |
| LEVEL OF HELP RECEIVED (FRIENDS OR RELATIVES)HOW MUCH HELP DOES THE PERSON RECEIVE FROM FRIENDS OR RELATIVES WITH THE MANAGEMENT OF THEIR DAILY STRESS? | Can 2403 |  |
|  |  |  |
| **□□□□□□□□□□□** **□** NOT KNOWN  None Very high help  NONE e.g. Receives no support.  VERY HIGH HELP e.g. Continuous support. | | |
| LEVEL OF HELP RECEIVED (SERVICES)HOW MUCH HELP DOES THE PERSON RECEIVE FROM LOCAL SERVICES WITH THE MANAGEMENT OF THEIR DAILY STRESS? | Can 2405 |  |
|  |  |  |
| **□□□□□□□□□□□** **□** NOT KNOWN  None Very high help  NONE e.g. Receives no support.  VERY HIGH HELP e.g. Coping strategies support group. | | |
| **LEVEL OF HELP NEEDED (SERVICES)** HOW MUCH HELP DOES THE PERSON NEED FROM LOCAL SERVICES WITH THE MANAGEMENT OF IS OR HER DAILY STRESS? | Can 2407 |  |
|  |  |  |
| **□□□□□□□□□□□** **□** NOT KNOWN  None Very high help  NONE e.g. No support needed.  VERY HIGH HELP e.g. Coping strategies support group. | | |

| APPROPRIATENESS OF CARE (SERVICES)DOES THE PERSON RECEIVE THE RIGHT TYPE OF HELP WITH MANAGING THEIR DAILY STRESS? | Can 2409 |  |
| --- | --- | --- |
|  |  |
| **QUANTITY**  **□□□□□□□□□□□** **□** NOT KNOWN  Not at all Entirely satisfied  satisfied | | |
| APPROPRIATENESS OF CARE (SERVICES)DOES THE PERSON RECEIVE THE RIGHT TYPE OF HELP WITH MANAGING THEIR DAILY STRESS? | Can 2411 |  |
|  |  |
| **QUALITY**  **□□□□□□□□□□□** **□** NOT KNOWN  Not at all Entirely satisfied  satisfied | | |
| USER’S PERSPECTIVE OF SERVICES REQUIRED | | |
| Thinking back about services received in managing your daily stress, tell us in a few words what you most appreciated. (Write down respondent’s spontaneously given information)  In your experience, what could be improved? (Write down respondent’s spontaneously given information) | | |

| 25. SOCIAL EXCLUSION | Assessments | |
| --- | --- | --- |
|  | *User* |  |
| DOES THE PERSON FEEL ACCEPTED IN THEIR SURROUNDINGS?*Do you have difficulty in getting accepted?**Do you feel discriminated against?* | Can 2501 |  |
|  |  |  |
| Please rate importance of current problem.  **□□□□□□□□□□□** **□** NOT KNOWN  No problem Very serious problem IF RATING IS *NO PROBLEM* OR *NOT KNOWN*, GO TO ITEM 26. | | |
| LEVEL OF HELP RECEIVED (FRIENDS OR RELATIVES)HOW MUCH HELP DOES THE PERSON RECEIVE FROM FRIENDS OR RELATIVES WITH BEING MORE READILY ACCEPTED IN THEIR SURROUNDINGS? | Can 2503 |  |
|  |  |  |
| **□□□□□□□□□□□** **□** NOT KNOWN  None Very high help  NONE e.g. Does not verify.  VERY HIGH HELP e.g. Direct support in front of discrimination. | | |
| LEVEL OF HELP RECEIVED (SERVICES)HOW MUCH HELP DOES THE PERSON RECEIVE FROM LOCAL SERVICES WITH BEING MORE ACCEPTED IN THEIR SURROUNDING? | Can 2505 |  |
|  |  |  |
| **□□□□□□□□□□□** **□** NOT KNOWN  None Very high help  NONE e.g. Offers no information on their rights.  VERY HIGH HELP e.g. Participates in intensive social integration program. | | |
| **LEVEL OF HELP NEEDED (SERVICES)** HOW MUCH HELP DOES THE PERSON NEED FROM LOCAL SERVICES WITH BEING MORE READILY ACCEPTED IN THEIR SURROUNDINGS? | Can 2507 |  |
|  |  |  |
| **□□□□□□□□□□□** **□** NOT KNOWN  None Very high help  NONE e.g. Needs no information.  VERY HIGH HELP e.g. Participates in intensive social integration program. | | |

| APPROPRIATENESS OF CARE (SERVICES)DOES THE PERSON RECEIVE THE RIGHT TYPE OF HELP WITH BEING MORE READILY ACCEPTED IN THEIR SURROUNDINGS? | Can 2509 |  |
| --- | --- | --- |
|  |  |
| **QUANTITY**  **□□□□□□□□□□□** **□** NOT KNOWN  Not at all Entirely satisfied  satisfied | | |
| APPROPRIATENESS OF CARE (SERVICES)DOES THE PERSON RECEIVE THE RIGHT TYPE OF HELP WITH BEING MORE READILY ACCEPTED IN THEIR SURROUNDINGS? | Can 2511 |  |
|  |  |
| **QUALITY**  **□□□□□□□□□□□** **□** NOT KNOWN  Not at all Entirely satisfied  satisfied | | |
| USER’S PERSPECTIVE OF SERVICES REQUIRED | | |
| Thinking back about services received to be more readily accepted in your surroundings, tell us in a few words what you most appreciated. (Write down respondent’s spontaneously given information)  In your experience, what could be improved? (Write down respondent’s spontaneously given information) | | |

| 26. INVOLVEMENT IN DECISIONS CONCERNING TREATMENT | Assessments | |
| --- | --- | --- |
|  | *User* |  |
| CAN THE PERSON GIVE HER OPINION CONCERNING THEIR TREATMENT?*Is the staff considering your preferences with regards to your treatment?* | Can 2601 |  |
|  |  |  |
| Please rate importance of current problem.  **□□□□□□□□□□□** **□** NOT KNOWN  No problem Very serious problem  **IF RATING IS *NO PROBLEM* OR *NOT KNOWN*, GO TO LAST SECTION.** | | |
| LEVEL OF HELP RECEIVED (FRIENDS OR RELATIVES)HOW MUCH HELP DOES THE PERSON RECEIVE FROM FRIENDS OR RELATIVES WITH ACHIEVING GREATER INVOLVEMENT IN THEIR TREATMENT? | Can 2603 |  |
|  |  |  |
| **□□□□□□□□□□□** **□** NOT KNOWN  None Very high help  NONE e.g. No support.  VERY HIGH HELP e.g. Unconditional support. | | |
| LEVEL OF HELP RECEIVED (SERVICES)HOW MUCH HELP DOES THE PERSON RECEIVE FROM FRIENDS OR RELATIVES WITH ACHIEVING GREATER INVOLVEMENT IN HIS/HER TREATMENT? | Can 2605 |  |
|  |  |  |
| **□□□□□□□□□□□** **□** NOT KNOWN  None Very high help  NONE e.g. Advice never solicited.  VERY HIGH HELP e.g. Opinion always listened to. | | |
| **LEVEL OF HELP NEEDED (SERVICES)** HOW MUCH HELP DOES THE PERSON NEED FROM FRIENDS OR RELATIVES WITH ACHIEVING GREATER INVOLVEMENT IN THEIR TREATMENT? | Can 2607 |  |
|  |  |  |
| **□□□□□□□□□□□** **□** NOT KNOWN  None Very high help  NONE e.g. No help needed.  VERY HIGH HELP e.g. Opinion always listened to. | | |

| APPROPRIATENESS OF CARE (SERVICES)DOES THE PERSON RECEIVE THE RIGHT TYPE OF HELP WITH ACHIEVING GREATER INVOLVEMENT IN THEIR TREATMENT? | Can 2609 |  |
| --- | --- | --- |
|  |  |
| **QUANTITY**  **□□□□□□□□□□□** **□** NOT KNOWN  Not at all Entirely satisfied  satisfied | | |
| APPROPRIATENESS OF CARE (SERVICES)DOES THE PERSON RECEIVE THE RIGHT TYPE OF HELP WITH ACHIEVING GREATER INVOLVEMENT IN THEIR TREATMENT? | Can 2611 |  |
|  |  |
| **QUALITY**  **□□□□□□□□□□□** **□** NOT KNOWN  Not at all Entirely satisfied  satisfied | | |
| USER’S PERSPECTIVE OF SERVICES REQUIRED | | |
| Thinking back about services received in achieving greater involvement in your treatment, tell us in a few words what you most appreciated. (Write down respondent’s spontaneously given information)  In your experience, what could be improved? (Write down respondent’s spontaneously given information) | | |

## C. MAIN SUPPORT CONSULTED

*List main staff consulted for each topic* ***during the last month****.*

*Specify their profession, their affiliated organization, the type of services offered by this organization, the length of the user/staff relationship (in months), the frequency of contacts (per month) as well as the modality and places of meeting.*

| **Main support consulted for :** | **Profession** | **Organization** | **Type of services** | **Since when?** | | **Frequency of contacts/month** | | | **Place of meeting** |
| --- | --- | --- | --- | --- | --- | --- | --- | --- | --- |
|  |  |  | *e.g. housing, support groups, crisis center* | |  | | **Face to face** | **Over the phone** | *e.g. services, at home or public space* |
| 1. Accommodation |  |  |  | |  | |  |  |  |
| 1. Food |  |  |  | |  | |  |  |  |
| 1. Looking after the home |  |  |  | |  | |  |  |  |
| 1. Self care |  |  |  | |  | |  |  |  |
| 1. Daytime activities |  |  |  | |  | |  |  |  |
| 1. Physical health |  |  |  | |  | |  |  |  |
| 1. Psychotic symptoms |  |  |  | |  | |  |  |  |
| 1. Information (about condition and treatment) |  |  |  | |  | |  |  |  |
| 1. Psychological distress |  |  |  | |  | |  |  |  |
| 1. Safety to self |  |  |  | |  | |  |  |  |
| 1. Safety to others |  |  |  | |  | |  |  |  |
| 1. Alcohol |  |  |  | |  | |  |  |  |
| 1. Drugs |  |  |  | |  | |  |  |  |
| 1. Company |  |  |  | |  | |  |  |  |
| 1. Intimate relationships |  |  |  | |  | |  |  |  |
| 1. Sexual expression |  |  |  | |  | |  |  |  |
| 1. Childcare |  |  |  | |  | |  |  |  |
| 1. Basic education |  |  |  | |  | |  |  |  |
| 1. Telephone |  |  |  | |  | |  |  |  |
| 1. Transport |  |  |  | |  | |  |  |  |
| 1. Money |  |  |  | |  | |  |  |  |
| 1. Social benefits |  |  |  | |  | |  |  |  |
| 1. Work |  |  |  | |  | |  |  |  |
| 1. Adaptation to everyday stress |  |  |  | |  | |  |  |  |
| 1. Social exclusion |  |  |  | |  | |  |  |  |
| 1. Involvement in decisions concerning treatment |  |  |  | |  | |  |  |  |

**D. ANSWER CART**

Dear Madam, Dear Sir,

I’ am about to ask you some questions related to your assessment on your situation. Please check on the following scales, the level that match the best with your situation **during the last month**.

1. **PROBLEM (S1- Needs)**

On this scale, level 0 means that you had no problem; level 10 means that you had very serious problem; level 5 (in the middle) means that your problem was moderate.

| **No problem** | | |  |  |  |  |  |  | **Moderate**  **problem** | | | | |  |  |  |  |  |  | **Very serious problem** | | |
| --- | --- | --- | --- | --- | --- | --- | --- | --- | --- | --- | --- | --- | --- | --- | --- | --- | --- | --- | --- | --- | --- | --- |
|  | 0 |  | 1 |  | 2 |  | 3 |  | 4 |  | 5 |  | 6 |  | 7 |  | 8 |  | 9 |  | 10 |  |
|  |  |  |  |  |  |  |  |  |  |  |  |  |  |  |  |  |  |  |  |  |  |  |
|  |  |  |  |  |  |  |  |  |  |  |  |
|  |  |  |  |  |  |  |  |  |  |  |  |  |  |  |  |  |  |  |  |  |  |  |

1. **HELP (received – wanted or needed)**

**Help received : 1) from friends or relatives (S2- Received relative)**

**2) from services (S2- Received service)**

On this scale, level 0 means that you did not receive any help; level 10 means that you did receive an important help (very high); level 5 (in the middle) means that the help you received was moderate.

| **No help** | | |  |  |  |  |  |  | **Moderate**  **help** | | | | |  |  |  |  |  |  | **Very high help** | | |
| --- | --- | --- | --- | --- | --- | --- | --- | --- | --- | --- | --- | --- | --- | --- | --- | --- | --- | --- | --- | --- | --- | --- |
|  | 0 |  | 1 |  | 2 |  | 3 |  | 4 |  | 5 |  | 6 |  | 7 |  | 8 |  | 9 |  | 10 |  |
|  |  |  |  |  |  |  |  |  |  |  |  |  |  |  |  |  |  |  |  |  |  |  |
|  |  |  |  |  |  |  |  |  |  |  |  |
|  |  |  |  |  |  |  |  |  |  |  |  |  |  |  |  |  |  |  |  |  |  |  |

**Wanted or needed help from services (S3- Help wanted or needed)**

On this scale, level 0 means that you did not want or need any help; level 10 means that you wanted a very important help; level 5 (in the middle) means that the help you wanted was moderately important.

| **No help wanted** | | |  |  |  |  |  |  | **Moderate**  **help wanted** | | | | |  |  |  |  |  |  | **Important help wanted** | | |
| --- | --- | --- | --- | --- | --- | --- | --- | --- | --- | --- | --- | --- | --- | --- | --- | --- | --- | --- | --- | --- | --- | --- |
|  | 0 |  | 1 |  | 2 |  | 3 |  | 4 |  | 5 |  | 6 |  | 7 |  | 8 |  | 9 |  | 10 |  |
|  |  |  |  |  |  |  |  |  |  |  |  |  |  |  |  |  |  |  |  |  |  |  |
|  |  |  |  |  |  |  |  |  |  |  |  |
|  |  |  |  |  |  |  |  |  |  |  |  |  |  |  |  |  |  |  |  |  |  |  |

1. **RELEVANCE OF HELP RECEIVED (amount - quality )**

**Amount of help (S4- Help Quantity)**

On this scale, level 0 means that the amount of help you did receive was not satisfying; level 10 means that the amount of help you did receive was entirely satisfying; level 5 (in the middle) means the amount of help you did receive was moderately satisfying.

AMOUNT

| **Not at all satisfying** | | |  |  |  |  |  |  | **Moderately**  **satisfying** | | | | |  |  |  |  |  |  | **Entirely satisfying** | | |
| --- | --- | --- | --- | --- | --- | --- | --- | --- | --- | --- | --- | --- | --- | --- | --- | --- | --- | --- | --- | --- | --- | --- |
|  | 0 |  | 1 |  | 2 |  | 3 |  | 4 |  | 5 |  | 6 |  | 7 |  | 8 |  | 9 |  | 10 |  |
|  |  |  |  |  |  |  |  |  |  |  |  |  |  |  |  |  |  |  |  |  |  |  |
|  |  |  |  |  |  |  |  |  |  |  |  |
|  |  |  |  |  |  |  |  |  |  |  |  |  |  |  |  |  |  |  |  |  |  |  |

**Quality of help (S4- Help Quality)**

On this scale, level 0 means that the quality of help you did receive was not satisfying; level 10 means that the quality of help you did receive was entirely satisfying; level 5 (in the middle) means the quality of help you did receive was moderately satisfying.

**QUALITY**

| **Not at all satisfying** | | |  |  |  |  |  |  | **Moderately**  **satisfying** | | | | |  |  |  |  |  |  | **Entirely satisfying** | | |
| --- | --- | --- | --- | --- | --- | --- | --- | --- | --- | --- | --- | --- | --- | --- | --- | --- | --- | --- | --- | --- | --- | --- |
|  | 0 |  | 1 |  | 2 |  | 3 |  | 4 |  | 5 |  | 6 |  | 7 |  | 8 |  | 9 |  | 10 |  |
|  |  |  |  |  |  |  |  |  |  |  |  |  |  |  |  |  |  |  |  |  |  |  |
|  |  |  |  |  |  |  |  |  |  |  |  |
|  |  |  |  |  |  |  |  |  |  |  |  |  |  |  |  |  |  |  |  |  |  |  |
